# Role of Arf GTPases in fungal morphogenesis and virulence

**DOI:** 10.1371/journal.ppat.1006205

**Published:** 2017-02-13

**Authors:** Hayet Labbaoui, Stéphanie Bogliolo, Vikram Ghugtyal, Norma V. Solis, Scott G. Filler, Robert A. Arkowitz, Martine Bassilana

**Affiliations:** 1 Université Côte d’Azur, CNRS, INSERM, iBV, Parc Valrose, Nice, France; 2 Los Angeles Biomedical Research Institute at Harbor-UCLA Medical Center, Torrance, CA, United States of America; Duke University School of Medicine, UNITED STATES

## Abstract

Virulence of the human fungal pathogen *Candida albicans* depends on the switch from budding to filamentous growth, which requires sustained membrane traffic and polarized growth. In many organisms, small GTPases of the Arf (ADP-ribosylation factor) family regulate membrane/protein trafficking, yet little is known about their role in fungal filamentous growth. To investigate these GTPases in *C*. *albicans*, we generated loss of function mutants in all 3 Arf proteins, Arf1-Arf3, and 2 Arf-like proteins, Arl1 and Arl3. Our results indicate that of these proteins, Arf2 is required for viability and sensitivity to antifungal drugs. Repressible *ARF2* expression results in defects in filamentous growth, cell wall integrity and virulence, likely due to alteration of the Golgi. Arl1 is also required for invasive filamentous growth and, although *arl1/arl1* cells can initiate hyphal growth, hyphae are substantially shorter than that of the wild-type, due to the inability of this mutant to maintain hyphal growth at a single site. We show that this defect does not result from an alteration of phospholipid distribution and is unlikely to result from the sole Golgin Imh1 mislocalization, as Imh1 is not required for invasive filamentous growth. Rather, our results suggest that the *arl1/arl1* hyphal growth defect results from increased secretion in this mutant. Strikingly, the *arl1/arl1* mutant is drastically reduced in virulence during oropharyngeal candidiasis. Together, our results highlight the importance of Arl1 and Arf2 as key regulators of hyphal growth and virulence in *C*. *albicans* and identify a unique function of Arl1 in secretion.

## Author summary

Virulence of the human fungal pathogen *Candida albicans* relies on the switch from budding to highly polarized hyphal growth. Sustained membrane traffic is critical for such polarized growth and for the secretion of virulence factors. Small G-proteins function as molecular switches required for a variety of cellular processes and the Arf (ADP-ribosylation factor) class of proteins, in particular, is critical for membrane traffic. To investigate the role of this class of proteins in *C*. *albicans*, we generated loss of function mutants in all 5 Arf/Arl (Arf like) proteins. Our results reveal that only Arf2 is required for viability and sensitivity to antifungal drugs. We also show that Arf2 and Arl1 are required for hyphal growth, with *arl1/arl1* hyphal filaments 2-fold shorter than the wild-type. While repressible *ARF2* expression results in pleiotropic defects, deletion of *ARL1* results in defects in restricting the site of growth to a single location. Finally, we show that *Δ/pTetARF2* and *arl1/arl1* mutants have drastically reduced virulence, with *ARL1* particularly critical for oropharyngeal candidiasis. Together, our results identify Arf2 and Arl1 as key regulators of membrane traffic, critical for *C*. *albicans* hyphal growth and virulence.

## Introduction

Signal-dependent morphology changes are crucial for the virulence of a range of plant and human fungal pathogens. These dramatic cell shape changes require plasma membrane and cell wall targeting of a number of proteins, together with the secretion of extracellular hydrolytic enzymes, critical for pathogenicity [1–3]. In all eukaryotes, membrane/protein trafficking to the plasma membrane is mediated by vesicular transport between different cellular compartments. Small GTPases of the Arf (ADP-ribosylation factor) and Rab (Ras-related in brain) families, which are part of the Ras superfamily, regulate each step of these processes [4–9]. These small GTPases cycle between an active GTP bound state and an inactive GDP bound state, regulated primarily by GEFs (Guanine nucleotide Exchange Factors) and GAPs (GTPase Activating Proteins). The importance of Rab GTPases during hyphal growth has been investigated in filamentous fungi, such as *Aspergillus nidulans* and *Neurospora crassa* (recently reviewed in [10, 11]). On the other hand, while a number of studies in *Saccharomyces cerevisiae* have shed light on the roles of Arf GTPases, little is known about their role in hyphal growth, a process characteristic of filamentous fungi that is absent in this organism.

Fungal infections kill more than a million people every year, with *Candida albicans* a major fungal pathogen of humans that accounts for ~10% of hospital-acquired bloodstream infections, with a mortality rate exceeding 30%. The success of *C*. *albicans* as a human fungal pathogen results in part from its ability to switch between different morphological states, in order to adapt to the diverse challenges of the human host [12–15]. The spatio-temporal regulation of exocytosis and endocytosis is likely to be crucial for growth and morphology changes [16, 17], and these two processes are critical for hyphal growth (reviewed in [18]). For example in *C*. *albicans*, during filamentous growth, secretory vesicles are clustered at the tip of the filament [19], at a structure called the Spitzenkörper, and the Golgi apparatus redistributes to the apex region [20, 21]. Interestingly, it was recently proposed that such Golgi polarization during hyphal growth is regulated by cAMP-PKA signaling *via* the Rab GAP Gyp1 [22]. Furthermore, endocytosis sites form a collar just below the tip of a growing hyphal filament [20, 23].

Mammalian Arf proteins have been divided into three classes based upon amino acid sequence identity [6, 24]; in *S*. *cerevisiae* and *C*. *albicans* only homologs of Class I and Class III members are present. These fungi have 5 Arf/Arl homologs compared to 26 in Human [6]. In contrast to *S*. *cerevisiae*, the role of Arf/Arl proteins is largely unknown in filamentous and dimorphic fungi. In *A*. *nidulans*, ArfB, the *S*. *cerevisiae* Arf3 homolog, is involved in polarized growth and endocytosis [25], as well as its homolog in *Magnaporthe oryzae*, Arf6, during asexual development [26]. In *C*. *albicans* a null mutant of the *S*. *cerevisiae* Arf GAP Gcs1 ortholog, *AGE3*, was shown to have increased antifungal drug sensitivity, reduced hyphal invasive growth and attenuated virulence in a mouse model of hematogenously disseminated candidiasis (HDC) [27, 28]. Here, we investigate the role of the complete set of Arf/Arl proteins in *C*. *albicans* external-signal mediated induced filamentous growth, cell wall integrity and virulence. Our results show that of the 5 Arf/Arl proteins, only Arf2 is essential for viability and drug sensitivity. On the other hand, Arl1 is required for hyphal growth maintenance and virulence during oropharyngeal candidiasis (OPC). Specifically, Arl1 is critical for restricting hyphal growth to a single site and our results suggest that this function of Arl1 is *via* regulation of protein secretion.

## Results

### Arf2 is required for viability and antifungal drug sensitivity

**Fig 1:**
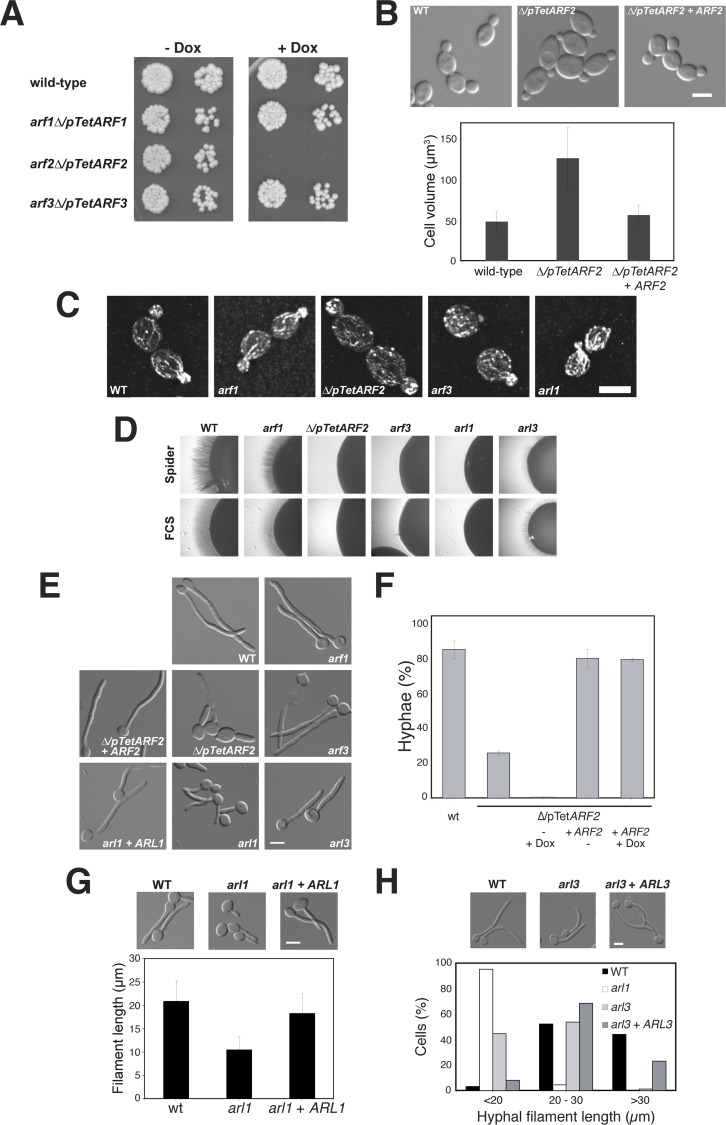
Arf2 and Arl1 are critical for hyphal invasive growth. A) *ARF2* is essential for viability. Exponentially growing cells from indicated strains were spotted on YEPD media with or without 20 μg/ml Dox and incubated for 2 days at 30°C. B) *Δ/pTetARF2* cells have an increased volume. Indicated strains were grown at 30°C in the absence of Dox and cell volume was measured. Error bars indicate the mean +/- the SD of 3 experiments, *n* = 60 cells each. C) *Δ/pTetARF2* cells have a polarized actin cytoskeleton. Indicated strains were grown in the absence of Dox and the actin cytoskeleton was visualized using Alexa 568-phalloidin. Note that *arf1/arf1*, *arf3/arf3, arl1/arl1* and *arl3/arl3* deletion strains are indicated in all figures as *arf1*, *arf3*, *arl1* and *arl3*. D) The *Δ/pTetARF2*, *arf3/arf3* and *arl1/arl1* mutants are defective in invasive filamentous growth. Indicated strains were grown, in the absence of Dox, on agar-containing YEPD with FCS or on Spider media and images were taken after 5 days. Similar results were observed in 2 independent experiments. E) The *Δ/pTetARF2* mutant is defective in hyphal growth in response to serum. Cells from the indicated strains were incubated with FCS for 90 min at 37°C, in the absence of Dox. F) Quantification of the *Δ/pTetARF2* mutant defect. The graph represents the percentage of hyphae in the indicated strains (mean 3 experiments, *n* = 150 cells each) after 90 min exposure to FCS at 37°C, in the presence or absence of Dox (0.005 μg/ml). The *∆/pTetARF2* mutant formed 25 ± 1.5% hyphae (compared to 74 ± 2.1% and 80 ± 5% for the WT and complemented strains, respectively), 56 ± 5.5% pseudohyphae (compared to 18 ± 3% and 13 ± 5% for the WT and complemented strains, respectively) and 19 ± 4% budding cells (compared to 8 ± 1% and 7 ± 1% in the WT and complemented strains, respectively). G and H) The *arl1/arl1* and, to a lesser extent, the *arl3/arl3* mutants have shorter hyphae. Filament length was measured in the indicated strains incubated for 90 min in the presence of FCS at 37°C. Error bars indicate the mean +/- the SD of 3 experiments, *n* = 40 cells each (G). The hyphal filament length distribution is illustrated from a typical experiment with the indicated strains.

S1A Fig shows the protein sequence alignment of *C*. *albicans* Arf1, Arf2, Arf3 and Arl1. Arl3, which is less similar to these four small G-proteins, is shown in S1B Fig, aligned with its *S*. *cerevisiae* and Human (ARFRP1) counterparts. Since in *S*. *cerevisiae* none of these genes is essential for viability [29, 30], we attempted to generate *C*. *albicans* strains in which both alleles were deleted. We succeeded in generating homozygous deletion mutants for all *ARF*/*ARL* genes except *ARF2*. Hence the tetracycline repressible promoter system was used to generate *arf2Δ/pTetARF2* mutants (hereafter referred to as *Δ/pTetARF2*) in which one copy of the gene was deleted and the Tet_off_ promoter was inserted upstream of the remaining copy, as described in S2A Fig. The *Δ/pTetARF2* mutant was verified by PCR of genomic DNA (S2B Fig) and Southern analyses (S2C Fig). The deletion mutants were verified by RT-PCR (S2E and S2F Fig). Fig 1A shows that Arf2 is required for viability, as the *Δ/pTetARF2* strain did not grow in the presence of the repressor doxycycline (Dox). Reintegration of a copy of *ARF2* into this *Δ/pTetARF2* strain restored viability in the presence of Dox (S4A Fig). In the absence of Dox, all *arf*/*arl* mutant strains grew similar to the WT strain, except for the *Δ/pTetARF2* strain, which exhibited a dramatic growth defect at 42°C (S3A Fig).

Strikingly, the size of the *Δ/pTetARF2* mutant cells, grown in the absence of Dox, was increased, with a ~ 2.5-fold larger volume compared to WT cells (Fig 1B). Addition of *ARF2* in the *Δ/pTetARF2* strain restored the cell size to that of the WT. Despite the significant increase in the *Δ/pTetARF2* cell volume, the growth rate of this strain was comparable to that of the WT strain. Furthermore, *Δ/pTetARF2* as well as *arf1/arf1*, *arf3/arf3* and *arl1/arl1* cells polarized their actin cytoskeleton similar to that of WT cells (Fig 1C), suggesting that there was not a general cell polarity defect in the *Δ/pTetARF2* strain. We did not observe a dramatic alteration in the morphology of the other *arf/arl* mutants, although the *arf3/arf3* cells did appear slightly rounder (Fig 1C). Together these data indicate that Arf2 is critical for viability and cell size regulation during budding growth, in contrast to *S*. *cerevisiae*. The different importance of *ARF1* and *ARF2* in *C*. *albicans* could result from the difference in the relative expression of each gene, as *ARF2* expression was > 10-fold higher, as determined by RNAseq analysis (S2G Fig).

S3B Fig and S4D Fig show that the *Δ/pTetARF2* mutant did not grow in the presence of fluconazole (Fluco) or caspofungin (Caspo) in the absence of Dox and this defect was complemented by reintroduction of *ARF2*. Reduced expression of the Golgi PI-4-kinase Pik1 resulted in a similar defect, which was due to an alteration of the distribution of the plasma membrane ABC multidrug transporter Cdr1 [20], however, this is not the case with the *Δ/pTetARF2* mutant (S3C Fig), similar to the *age3/age3* mutant [28]. The *Δ/pTetARF2* mutant, and to a lesser extent *arl1/arl1* mutant, also had reduced growth on the cell wall perturbants congo red (CR) and calcofluor white (CFW) (S3B Fig). In both cases, the defect was complemented by the addition of the respective gene (S4C and S4E Fig). In contrast, deletion of the other *ARF/ARL* genes resulted in little to no growth alteration in the presence of cell wall stress or antifungal drugs (S3B Fig).

### Arf2 and Arl1 are critical for invasive filamentous growth and virulence

We next investigated whether Arf/Arl were required for *C*. *albicans* filamentous growth and Fig 1D shows that *Δ/pTetARF2*, *arf3/arf3*, *arl1/arl1*, and to a lesser extent *arl3/arl3* mutants were dramatically reduced in invasive growth in response to fetal calf serum (FCS) or the carbon source-poor Spider medium. These defects were complemented by reintroduction of the respective gene (S4B and S4F Fig). We also observed that the *Δ/pTetARF2* and *arl1/arl1* mutants, but not the *arf3/arf3* mutant, had reduced hyphal growth in response to FCS in liquid media (Fig 1E). After 90 min in the presence of FCS, *Δ/pTetARF2* mutant cells formed less than 30% hyphae, compared to approximately 80% for the WT and complemented strains (Fig 1F). Growth of this strain in low levels of Dox (0.005 μg/ml), a concentration that does not substantially affect budding growth, further increased the hyphal growth defect, with less than 1% hyphae formed. We observed substantially less *ARF2* mRNA in this growth condition, in contrast to the absence of Dox, where *ARF2* mRNA transcript levels were similar to that of the wild-type (S2D Fig). It is likely that there is suboptimal function of Arf2 in the *∆*/*pTetARF2* strain (in the absence of Dox), as the defect of the *∆/pTetARF2* is complemented by addition of a copy of *ARF2*, suggesting that *ARF2* regulation *via* its promoter is critical. On the other hand, the *arl1/arl1* mutant formed filaments that were ~2-fold shorter (Fig 1G), and the *arl3/arl3* mutant filaments were an intermediate length between that of WT and *arl1/arl1* strains (Fig 1H). These defects were all complemented by reintroduction of the respective gene (Fig 1E, 1F, 1G and 1H). Together our results show that both Arf2 and Arl1 are critical for invasive and filamentous growth.

**Fig 2:**
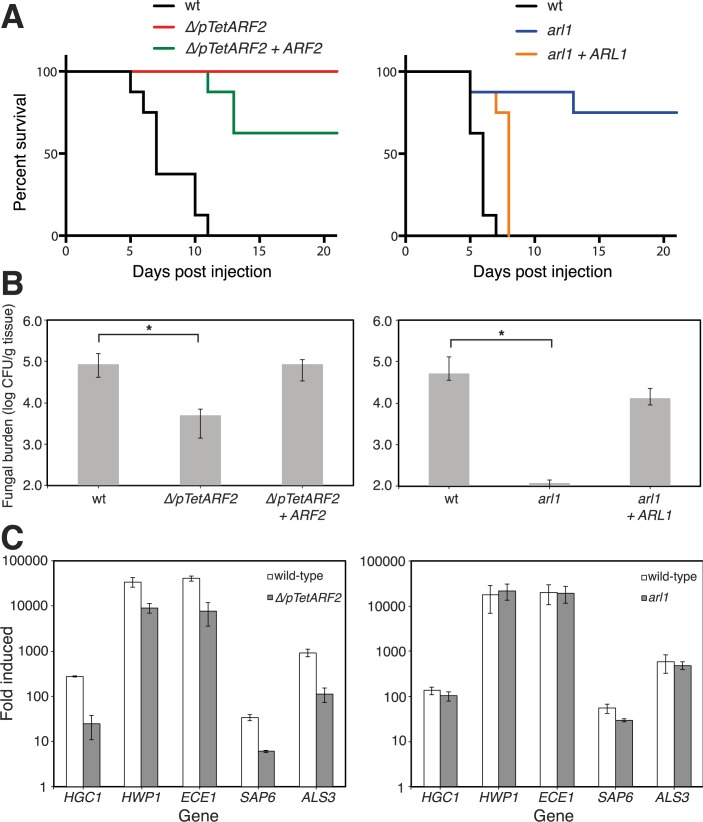
Arf2 and Arl1 are required for virulence. A) The *Δ/pTetARF2* and *arl1/arl1* mutants have attenuated virulence in a mouse model of hematogenously disseminated candidiasis. Balb/C mice (*n* = 8) were injected with an inoculum (5 x 10^5^ cells) of the indicated strains and the survival was assessed. *Δ/pTetARF2*, p < 0.0001 and 0.06 compared to WT and complemented strains, respectively; *arl1/arl1*, p = 0.0009 and 0.003 compared to WT and complemented strains, respectively. B) Arl1 is critical for oropharyngeal candidiasis. The oral fungal burden of mice infected with the indicated strains was measured after 5 days of infection. Results are in the medians ± interquartile ranges of 8 mice per strain. *, p = 0.002 and 0.006 compared to WT for *Δ/pTetARF2* and *arl1/arl1*, respectively, (p = 0.0006 and 0.005 compared to their respective complemented strains). C) Hyphal specific gene (HSGs) induction is reduced in *Δ/pTetARF2*, but not in the *arl1/arl1* mutant. Transcript levels determined by qRT-PCR of *HGC1*, *HWP1*, *ECE1*, *SAP6* and *ALS3* are normalized to the *ACT1* transcript level. Fold induction of HSGs after incubation of cells for 120 min in FCS at 37°C is shown. Fold induction is the normalized transcript level at 120 min divided by the normalized transcript level at time zero. The mean values of two independent experiments are shown with bars indicating values of each experiment.

We then examined the virulence of *Δ/pTetARF2* and *arl1/arl1* mutants in two murine infection models, hematogenously disseminated candidiasis (HDC) and oropharyngeal candidiasis (OPC). In the HDC model, 100% and 75% of mice infected with *Δ/pTetARF2* and *arl1/arl1*, respectively, survived 3 weeks after injection, while all of the mice infected with the WT strain died within 11 days (Fig 2A). In the OPC model (Fig 2B), *Δ/pTetARF2* and *arl1/arl1* mutants were also substantially less virulent than the WT, with the oral fungal burden of the infected mice reduced by ~30-fold and greater than 300-fold, respectively. Histological analyses of the infected tongues (S5A Fig) revealed that the *Δ/pTetARF2* mutant nonetheless formed filaments *in vivo*. The dramatic reduction of *arl1/arl1* cell infection of the oropharyngeal cavity appears to be niche-specific as the fungal burden in kidney and liver from the HDC mice was similar between this mutant strain and the WT (S5B Fig). Intriguingly, the fungal burden in the brain of mice infected with the *arl1/arl1* mutant was substantially higher than those infected with the WT or complemented strain, suggesting a significant increase in tropism for the brain (S5B Fig). As *arf3/arf3* and *arl3/arl3* were somewhat defective for invasive growth and/or cell wall integrity, these strains were also tested for virulence. They were essentially as virulent as the WT in the two murine models (*i*.*e*, in OPC, the fungal burdens were similar and in HDC, 100% of mice infected with *arf3/arf3* and *arl3/arl3* died within 8 and 13 days post injection, respectively, compared to 7 days for WT). All in all these data indicate that both *ARF2* and *ARL1* are required for virulence, with *ARL1* particularly critical for OPC.

*C*. *albicans* responds to cues, such as the presence of FCS, by inducing a number of hyphal specific genes (HSG), critical for virulence. The induction of all HSGs examined–including the hypha-specific G1 cyclin encoding gene *HGC1* [31] was reduced ~ 4 to 10-fold in *Δ/pTetARF2* cells compared to WT (Fig 2C). In contrast, HSG induction was not altered in *arl1/arl1*, except *SAP6*, which was slightly reduced (~ 2-fold). Together these results indicate that Arf2 and Arl1 have distinct roles during hyphal growth and virulence.

### Arf2, but not Arl1, is critical for the number of Golgi cisternae

**Fig 3:**
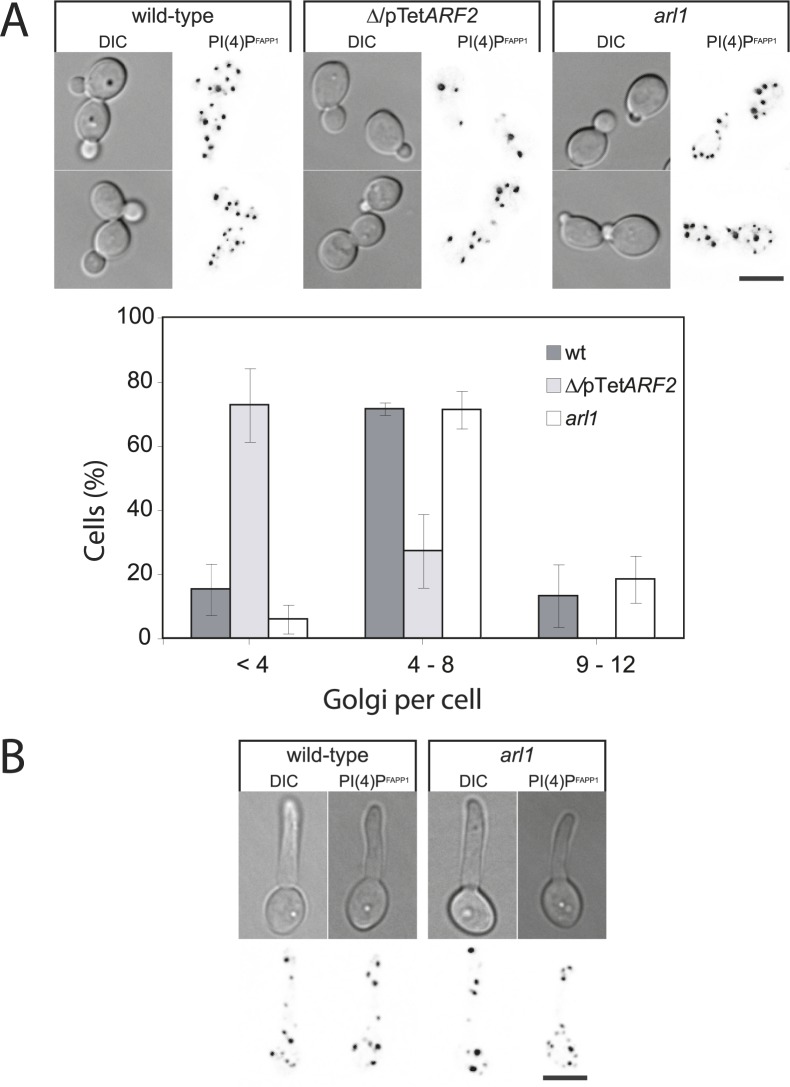
Arf2, but not Arl1, is required for late Golgi. A) The number of Golgi cisternae is altered in the *Δ/pTetARF2*, but not in the *arl1/arl1* mutant. DIC and maximum projection fluorescence images (21 deconvolved z-sections) of representative cells of indicated strains expressing FAPP1^[E50A,H54A]^GFP, are shown with an inverted LUT. The number of Golgi cisternae per cell was determined from maximum projections of deconvolved images (21 z-sections) of indicated strains. Error bars, in the graph, indicate the mean +/- the SD of 3 experiments, *n* = 30 cells and ~300 punctae each. B) The distribution of Golgi cisternae is not altered in the *arl1/arl1* hyphal cells. WT and *arl1/arl1* cells expressing *FAPP1*^*[E50A*,*H54A]*^*GFP*, were grown in FCS for 45 and 90 min, respectively. DIC and maximum projection fluorescence images (21 deconvolved z-sections) of representative cells are shown with an inverted LUT.

It is possible that the *Δ/pTetARF2* and/or *arl1/arl1* defects result from an alteration of the Golgi. In *S*. *cerevisiae*, enlarged late Golgi cisternae were observed in an *arf1/arf1* mutant [32]. Using the Four-phosphate-adaptor protein PH domain mutated for the Arf binding site, FAPP1^[E50A,H54A]^GFP [20], which specifically binds PI(4)P at the late Golgi, we determined that the number of Golgi cisternae in the *Δ/pTetARF2* mutant was substantially reduced compared to that of the WT and the *arl1/arl1* strains (Fig 3A), with 3.0 ± 1.3 Golgi cisternae per cell in *Δ/pTetARF2* cells compared to 5.8 ± 2.2 in the WT and 6.6 ± 2.0 in the *arl1/arl1* strains (*n* = 85–90 cells per strain). In contrast, the distribution and apparent morphology of the Golgi was similar in WT and *arl1/arl1* hyphal filaments (Fig 3B). These results indicate that Arf2, but not Arl1, is required for maintaining the number of Golgi cisternae.

### *C*. *albicans* Arl1 is a *bona-fide* GTP binding protein that localizes to the late Golgi

**Fig 4:**
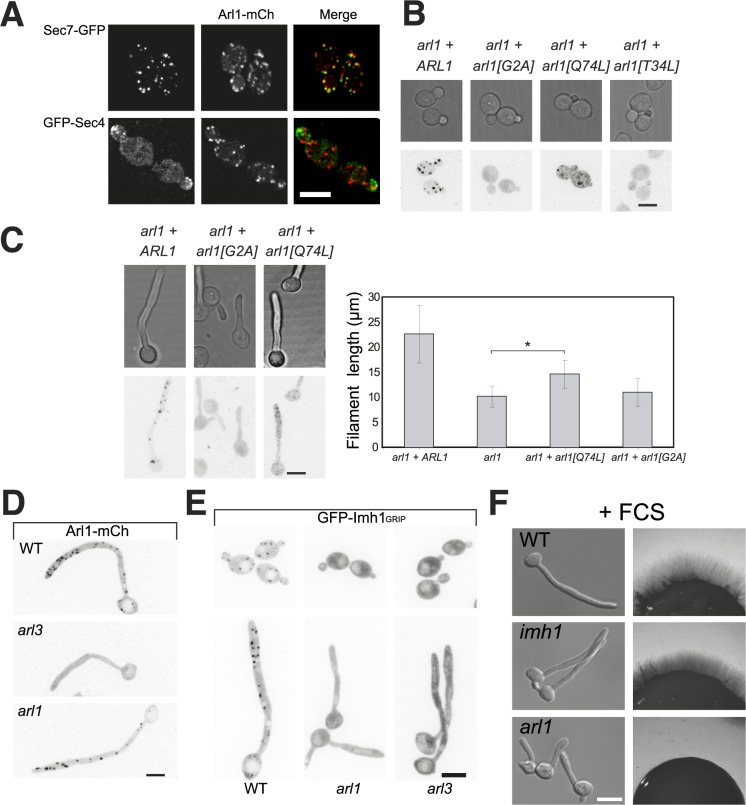
The conserved Arl3/Arl1/Imh1 pathway is not required for hyphal growth. A) Arl1 is associated with the late Golgi. Maximum projections of 21 deconvolved z-sections of representative cells expressing Arl1-mCherry together with Sec7-GFP (top) or GFP-Sec4 (bottom). GFP and mCherry signals were acquired simultaneously. B-C) Arl1 behaves as a GTPase. Maximum projections of 21 deconvolved z-sections of representative *arl1/arl1* cells expressing Arl1-mCherry and the mutated versions [G2A], [Q74L] and [T34L] during budding (B) and FCS-induced hyphal growth (C). Arl1[Q74L] can partially rescue the *arl1/arl1* hyphal growth defect. Error bars, in the graph right panel, indicate the mean +/- the SD of the average filament lengths of ~ 25 cells for each strain. *, p < 0.0001. D-E) Arl3 affects Arl1 and Imh1 distribution. Maximum projections of 21 deconvolved z-sections of representative indicated cells expressing Arl1-mCherry (D) or the GRIP domain of Imh1 fused to GFP (E), after 90 min FCS incubation. F) Imh1 is not required for hyphal invasive growth. Cells from the indicated strains were incubated with FCS for 90 min (left panel) or on agar-containing YEPD with FCS and images were taken after 5 days (right panel). Note that the *imh1/imh1* deletion strain is indicated as *imh1*. Similar results were observed in 2 independent experiments.

To further characterize *C*. *albicans* Arl1, we mutated the putative myristoylation site (Arl1[G2A], [33]) and generated putative GTP binding-defective (Arl1[T34L]) and GTP hydrolysis-deficient (Arl1[Q74L]) mutants [34]. These mutated forms were fused with yemCherry (Arl1-mCh) and expressed in the *arl1/arl1* mutant as the sole copy. We first determined that Arl1-mCh complemented the *arl1/arl1* hyphal growth defect (S4G Fig) and localized as punctae (Fig 4A–4D). Comparison of the localization of this functional Arl1-mCh to that of the late Golgi marker Sec7-GFP in WT cells indicates that ~ 80% of Arl1 containing punctae had Sec7 signal (*n* = 50 cells) with < 15% of Arl1 containing punctae having the secretory vesicle marker Sec4 (*n* = 35 cells), indicating that Arl1 is associated with the late Golgi (Fig 4A). Arl1[Q74L] also localized as punctae, although there was more diffuse signal than with Arl1; both Arl1[T34L] and Arl1[G2A] were cytosolic (Fig 4B), indicating that the inactive and non-myristoylated GTPase are not targeted to the late Golgi. Arl1[T34L] appeared to be deleterious as only 7.8 ± 1.7% hyphae formed in the presence of FCS, compared to 87.6 ± 1.3%, 78.6 ± 4.4% and 73.8 ± 1.5% for Arl1, Arl1[G2A] and Arl1[Q74L], respectively. Furthermore, Fig 4C shows that Arl1[Q74L] partially complemented *arl1/arl1* hyphal growth defect, in contrast to Arl1[G2A] that, as expected, did not complement this defect. Together these data establish that *C*. *albicans* Arl1 is a *bona-fide* GTP binding protein, localized to the late Golgi, similar to Arl1 in other organisms [35, 36].

### Arl1-dependent Golgin recruitment is not required for invasive filamentous growth

In *S*. *cerevisiae*, it was proposed that Arl3 recruits Arl1, which in turn recruits the sole yeast GRIP-domain golgin Imh1, a coiled-coil tethering protein [36, 37], raising the question of whether such a pathway exists in *C*. *albicans*. Using Arl1-mCh, little to no Arl1 punctae were observed in *arl3/arl3* cells compared to the WT (Fig 4D), indicating that Arl3 also facilitates Arl1 recruitment to the Golgi and/or stability at this organelle in *C*. *albicans*. Nonetheless, compared to Arl1, Arl3 appears less important for hyphal growth and virulence, suggesting that Arl1 has additional functions. To test genetically whether these two Arl proteins function in different pathways, we generated a double deletion mutant (S2F Fig) that is viable and does not exhibit additional hyphal growth defects compared to *arl1/arl1* alone (hyphal length of 12.8 ± 0.3 μm, compared to 12.4 ± 0.7 μm for the *arl1/arl1* mutant, determined as in Fig 1G), indicating that *arl1/arl1* and *arl3/arl3* do not exhibit a synthetic phenotype. We next generated a fusion between the GRIP domain of Imh1 (171 aa) and GFP, as previously described [36]. While this fusion protein localized as punctae both in WT budding and hyphal cells, little to no punctae were observed in *arl1/arl1* and *arl3/arl3* budding and hyphal cells (Fig 4E). These data suggest that the Arl3, Arl1, Imh1 pathway exists in *C*. *albicans* and raises the possibility that hyphal growth is partly regulated *via* this pathway. To test this, we generated an *IMH1* deletion mutant (S6A Fig). Fig 4F and S6B Fig show that hyphal invasive growth and sensitivity to cell wall perturbants were identical in *imh1/imh1* and WT cells, suggesting that the *arl1/arl1* defects are unlikely to result from misregulation *via* Imh1.

### Arl1 is required for polarized growth

**Fig 5:**
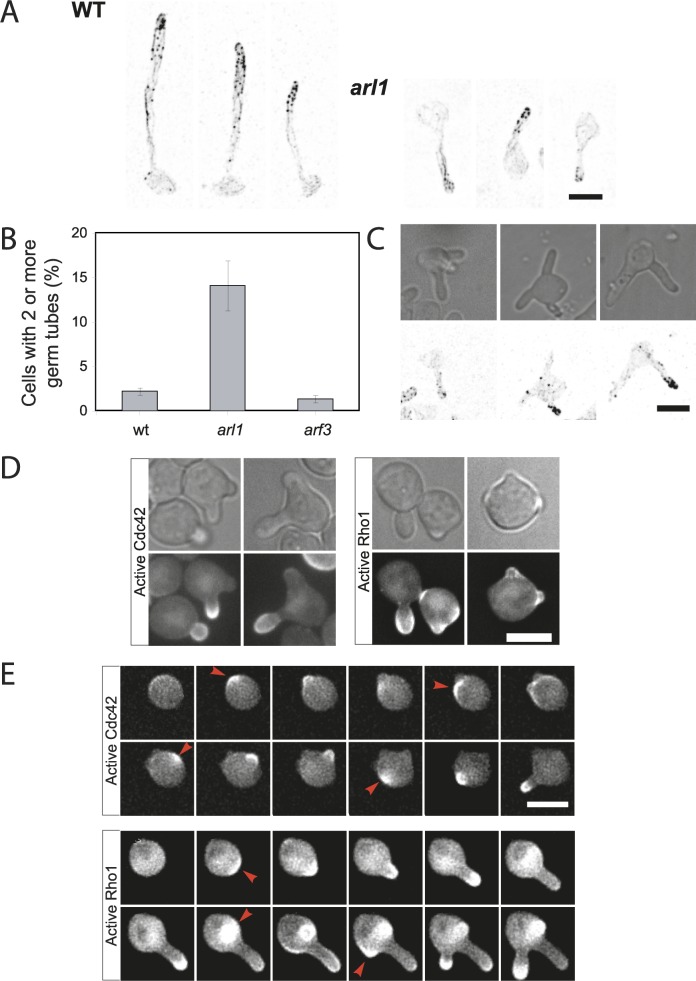
Arl1 is required for restricted polarized growth in hyphae. A) Actin cytoskeleton is polarized in *arl1/arl1* hyphae. WT and *arl1/arl1* cells were incubated in the presence of FCS for 90 min, prior to Alexa 568-phalloidin staining. B) The *arl1/arl1* mutant has an increased proportion of cells with 2 or more germ tubes. WT, *arl1/arl1* and *arf3/arf3* cells were incubated for 90 min in the presence of FCS and cells with 2 or more germ tubes were counted. Error bars indicate the mean +/- the SD of 4 experiments, *n* = 500 cells. C) Actin is polarized in only one of the germ tubes in *arl1/arl1* mutant. Cells were incubated for 90 min in the presence of FCS and the actin cytoskeleton was visualized using Alexa568-phalloidin. DIC and maximum projections (21 deconvolved z-sections) of representative cells are shown. D) Active Cdc42 is associated with only one of the germ tubes in the *arl1/arl1* mutant. *arl1/arl1* cells expressing a reporter for active Cdc42 or active Rho1, *i*.*e* CRIB-GFP or GFP-RID, respectively, were incubated for 45 min in the presence of FCS. DIC and sum projections (21 deconvolved z-sections) of representative cells are shown. E) Active Cdc42 is recruited sequentially to multiple sites. Time lapse of *arl1/arl1* cells expressing reporters for active Cdc42 and active Rho1, incubated in the presence of FCS. Images, taken every 5 min, are sum projections (21 deconvolved z-sections) and arrows indicate the sites of active Cdc42 or active Rho1 recruitment.

We postulated that the shorter *arl1/arl1* hyphae were either due to a reduced growth rate or a delay in germ tube initiation. First, we verified that actin was correctly polarized in the shorter *arl1/arl1* filaments (Fig 5A). Quantitation also revealed that in addition to forming shorter filaments, an increased number of *arl1/arl1* cells had multiple germ tubes (Fig 5B). Strikingly, we observed 14.1 ± 2.8% of *arl1/arl1* cells with two or more germ tubes, compared to 2.2 ± 0.4% for the WT, after 90 min in FCS. Given that in *S*. *cerevisiae* and *A*. *nidulans* the Arf3 homolog regulates polarized growth [25, 38, 39], we also analyzed this mutant and found that the percentage of cells with two germ tubes was identical to the WT, suggesting that this phenotype is Arl1 specific. In the *arl1/arl1* cells with multiple germ tubes, the actin cytoskeleton was only observed in one germ tube, indicating that they form sequentially (Fig 5C). Together these results suggest that Arl1 is important for restricting the growth to a single site.

To directly visualize sites of ongoing growth in *arl1/arl1* we used a CRIB-GFP reporter [40] to follow the distribution of active Cdc42 in cells with multiple germ tubes. As observed with F-actin, active Cdc42 was only associated with one germ tube at a time in the *arl1/arl1* mutant (Fig 5D) and time-lapse microscopy revealed that it was recruited sequentially to different sites (Fig 5E) in all of the cells with multiple germ tubes (~ 20% of the cells). In contrast to this sequential recruitment of active Cdc42, we observed that active Rho1 [40] appeared to linger at a growth site after it was abandoned and, in many instances, this active GTPase localizes to more than one site (Fig 5D and 5E). These results indicate that reduced germ tube length results in part from an inability to restrict growth to a single site in *arl1/arl1* cells. Consistent with this proposal, the filament extension rate in this mutant was only slightly reduced compared to that of WT (14 ± 2 μm/h in *arl1/arl1* compared to 18 ± 2 μm/h in WT). Together these results indicate a unique role for Arl1 in *C*. *albicans;* it is critical for restricting polarized growth to a single site.

### Phosphatidylserine distribution is not altered in the *arl1/arl1* mutant

**Fig 6:**
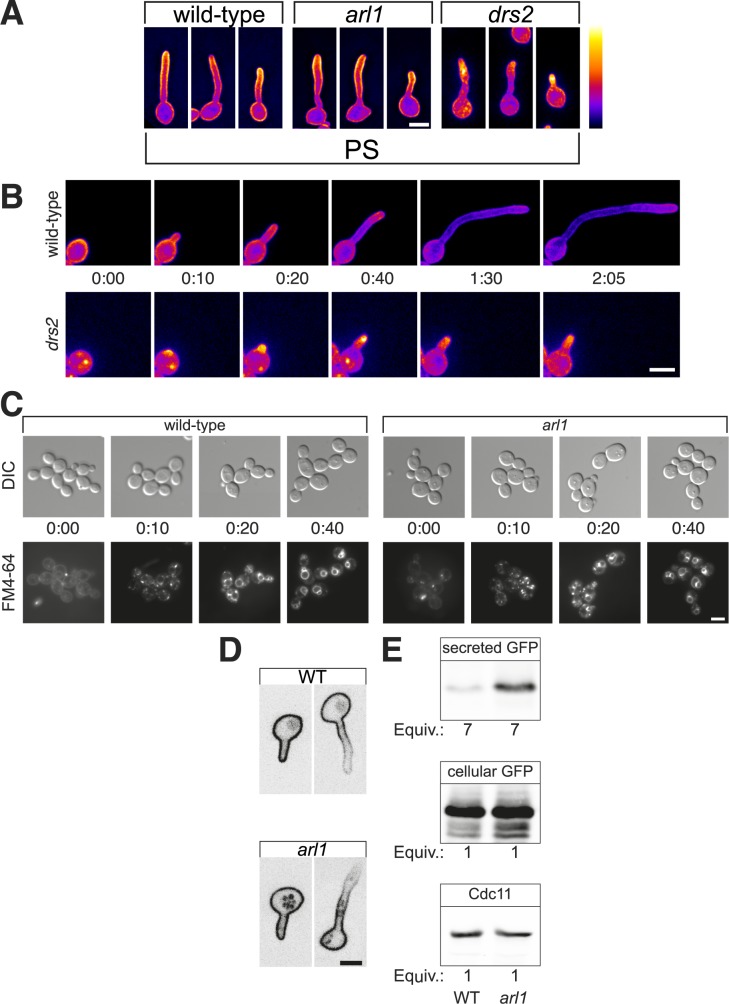
Arl1 regulates secretion. A) Distribution of PS is not altered in *arl1/arl1* mutant during hyphal growth. WT, *arl1/arl1* and *drs2/drs2* cells expressing the PS reporter (GFP-LactC2) were induced in the presence of FCS for 45 and 90 min, respectively and sum projections of representative images are shown. A false color look up table (LUT) is used to highlight signal intensity. Note that the *drs2/drs2* deletion strain is indicated as *drs2*. B) Distribution of PS and hyphal growth is altered in the *drs2/drs2* mutant. Time course of indicated strains expressing GFP-LactC2, incubated in the presence of FCS. Images, taken every 5 min, are sum projections (21 z-sections). C) FM4-64 endocytosis is not altered in the *arl1/arl1* mutant. WT and *arl1/arl1* cells were incubated with FM4-64 on ice for 40 min (time 0:00), and subsequently transferred to 30°C to initiate endocytosis, for the indicated times. Cells were visualized by fluorescence microscopy with the respective DIC images shown. D) Distribution of Phr2 is altered in the *arl1/arl1* mutant. WT and *arl1/arl1* cells, expressing yemCherry-Phr2 were grown in the presence of FCS for 45 and 90 min, respectively. Central Z sections of representative images are shown. E) The *arl1/arl1* mutant has increased secretion. WT and *arl1/arl1* cells, expressing HWP_ss_-GFP, were pelleted and BSA (100 μg/mL) was added to the supernatant prior to TCA (10%) precipitation. Following centrifugation, precipitated proteins were washed twice with ice-cold acetone, then analyzed by SDS/PAGE and immunoblotting. The amount of secreted Hwp1_ss_-GFP in the WT and *arl1/arl1* mutant strains was normalized using Cdc11 and the total cellular GFP signal, quantified from the cell pellet. The equivalents (Equiv.) of cells and culture supernatants are indicated; 7 times more culture supernatant was analyzed compared to cells.

Cdc42 distribution, hence polarized growth, requires phospholipids. For example, in *S*. *cerevisiae*, phosphatidylserine (PS) is required for the correct localization of Cdc42 [41]. In this organism, Arl1 regulates the activity of the PS flippase Drs2 [42], and to investigate the possibility that the *arl1/arl1* defect results from PS misregulation, we generated a Lact-C2 reporter similar to that described in *S*. *cerevisiae* [41, 43], to visualize PS in an *arl1* mutant. Fig 6A shows that PS was distributed similarly at the plasma membrane and enriched in the filament in WT and *arl1/arl1* cells. In contrast, PS distribution was altered in a *drs2/drs2* deletion mutant, *i*.*e* the PS reporter was less associated with the plasma membrane and appeared as internal punctae, enriched at the germ tube apex of these cells (Fig 6A and 6B). This *drs2/drs2* mutant was unaffected for budding growth, but showed a defect in FCS-dependent hyphal growth, with only 3.2 ± 2.6% of hyphae generated compared to 84.2 ± 0.9% in the WT strain. This *drs2/drs2* mutant (S6C Fig) was also hypersensitive to fluconazole, but not to CFW or CR, compared to the *arl1/arl1* mutant (S6D Fig). Time-lapse microscopy revealed that the *drs2/drs2* mutant could initiate germ tube formation but was unable to maintain hyphal growth (Fig 6B) and, unlike the *arl1/arl1* mutant, did not form an increased number of cells with multiple germ tubes (3.7 ± 0.4%, compared to 2.8 ± 0.7% and 16.4 ± 0.7%, for the WT and the *arl1/arl1* mutant, respectively, determined as in Fig 5B). In addition, overexpression of *DRS2* (S6C Fig) did not complement the filamentous growth defect in the *arl1/arl1* mutant (hyphal length of 11.5 ± 0.5 μm, compared to 11.4 ± 0.6 μm for the *arl1/arl1* mutant, determined as in Fig 1G). Together these results indicate that Drs2 is critical for hyphal growth and PS distribution, and suggest that the *arl1/arl1* polarized growth defect is not due to an altered PS distribution.

### The *arl1/arl1* mutant polarized growth defect results from altered secretion

A balance between exocytosis and endocytosis is likely to be important for sustained polarized growth and very recent work in *S*. *cerevisiae*, in particular, shows that the late stage of exocytosis is important for regulating endocytosis [16, 17]. In *C*. *albicans*, a exocyst subunit mutant (*SEC3* deletion) is viable yet it has shorter hyphae that bulge at the apex [44] and, similarly, expression of a phosphomimetic version of Sla1, a key component of the endocytic machinery, results in impaired endocytosis and defective hyphal growth [45]. Arf proteins have been implicated in endocytosis, both in *S*. *cerevisiae* [46] and *A*. *nidulans* [25]. In *C*. *albicans*, an *age3/age3* mutant is defective in endocytosis of the lipophilic dye FM4-64 [28]. We examined endocytosis in *arl1/arl1* cells by following FM4-64 uptake and observed that over 80% of the vacuoles were labeled in both *arl1/arl1* and WT cells after 30 min incubation (Fig 6C), indicating that Arl1 is not critical for endocytosis. We next investigated the exocytosis capacity in *arl1/arl1* cells by examining the distribution of Phr2, a GPI-anchored cell wall protein homologous to *S*. *cerevisiae* Gas1p, which is mislocalized in a *S*. *cerevisiae arl1* mutant [47]. As shown in Fig 6D, Phr2 distribution was similar at the plasma membrane in *C*. *albicans* hyphal WT and *arl1/arl1* cells. Nonetheless 79 ± 10% of *arl1/arl1* cells also had internal signal, compared to 7 ± 5% of WT cells (*n* = 40–50 cells), indicative of some mistargeting of Phr2 in the absence of Arl1. In contrast, the distribution of Cdr1, a 12 TMD multidrug transporter of the ABC superfamily, was not altered in *arl1/arl1*, compared to WT cells (S3C Fig). Using as a reporter a protein consisting of the Hwp1 signal sequence fused to GFP (Hwp1_ss_-GFP) [20], we investigated secretion in *arl1/arl1* cells. Fig 6E shows an immunoblot of the culture supernatants of WT and *arl1/arl1* cells expressing Hwp1_ss_-GFP. Strikingly, secretion of Hwp1_ss_-GFP, normalized for the total cellular GFP and Cdc11, was increased by 7.4 ± 1.3-fold in *arl1/arl1* cells compared to the WT (*n* = 3), suggesting that Arl1 is important for regulation of secretion. Together, these results suggest that the polarized growth defect in the *arl1/arl1* mutant results from unregulated secretion.

## Discussion

To determine the role of the Arf proteins in *C*. *albicans* development and virulence, we generated loss of function mutants in the 5 Arf/Arl proteins. Our results show that of these proteins, only Arf2 was required for viability and antifungal drug sensitivity. Furthermore, we demonstrated that Arf2 and Arl1 are required for invasive hyphal growth and critical for virulence using different infection assays. Our data also indicate that of the 5 Arf/Arl proteins, Arl1 has a unique function in secretion, which would impact hyphal growth maintenance.

The functions of Arf proteins are largely unknown in filamentous fungi and fission yeast, in contrast to the budding yeast *S*. *cerevisiae* and our results show that the relative importance of the conserved Arf proteins differ in fungi, highlighting the importance of such a study. As an example, in *S*. *cerevisiae*, Arf3, the homolog of human Arf6, is required for polarized growth [38] and actin cytoskeleton organization [48, 49], as well as endocytosis *via* modulation of plasma membrane PI(4,5)P_2_ levels [50]. Similarly in *A*. *nidulans*, the Arf3 homolog, ArfB, is involved in polarized growth and endocytosis [25]. Here we show that in the absence of *ARF3 C*. *albicans* cells still polarize their actin cytoskeleton and are able to form hyphae similar to the WT strain. In addition, while *arf3/arf3* cells exhibited reduced invasive growth, as reported for *S*. *cerevisiae* [51], there was little to no difference in virulence of this mutant compared to the WT strain in two different murine candidiasis models, indicating that Arf3 plays a minor role in this human fungal pathogen.

We show that Arf2 and Arl1 are important for invasive filamentous growth and virulence. In particular, the *arl1/arl1* mutant exhibits shorter hyphae, and attenuated virulence. Unfortunately, we could not assess if *arl1/arl1* hyphae were also significantly shorter than those of the WT *in vivo*. The histopathology images of the tongues, however, showed that both *arl1/arl1* and *Δ/pTetARF2* mutants were able to form hyphae to some extent, consistent with our *in vitro* data. The virulence of the *age3/age3* mutant was reduced in a HDC murine model and histopathology images of the kidneys showed that this mutant was also able to form hyphae *in vivo* [27]. Hence, *age3/age3*, *Δ/pTetARF2* and *arl1/arl1* mutants all showed reduced virulence, yet were able to form hyphae, suggesting hyphal formation *per se* is not the basis for the virulence defect in these mutants, yet some aspect of hyphal growth maintenance may be important.

Our data indicate that the integrity of the Golgi in *arl1/arl1* does not appear to be affected, in contrast to *Δ/pTetARF2* or the PI-4-kinase mutant *pik1* [20], suggesting that the late Golgi associated protein Arl1 is not critical for the maintenance of Golgi cisternae number or size. In *S*. *cerevisiae*, Arl1 was shown to interact with a number of proteins involved in membrane recycling, including Arl3 [36, 37] and the Golgin Imh1 [52], which were reported to function upstream and downstream of Arl1, respectively. Very recently, the protein kinase Env7 was shown to functionally interact with Imh1, and potentially Arl1, in *C*. *albicans* [53]. Although we show that an Arl3-Arl1-Imh1 pathway is likely conserved in *C*. *albicans*, our results also show that this pathway is not critical during filamentous growth and virulence. Indeed, Arl3 is not required for *C*. *albicans* virulence and the *arl3/arl3* filamentous growth defect is marginal compared to that of *arl1/arl1*. In addition, deletion of *IMH1* does not alter filamentous growth. Arl1 was also shown to interact with Vps53/Vps54, components of the Golgi associated retrograde protein (GARP) complex [54]. Interestingly, *C*. *albicans vps51* and *vps53* deletion strains had attenuated virulence in a mouse model of HDC and increased brain tropism [55], similar to *arl1/arl1*, suggesting that Arl1 may interact with the GARP complex. Whether the increased tropism for the brain of the *arl1/arl1* mutant is due to increased trafficking to the brain or enhanced growth within the brain remains to be determined.

What might be the role of Arl1 during *C*. *albicans* hyphal growth? Our results indicate that Arl1 is critical for cell polarity maintenance. In *S*. *cerevisiae*, phosphatidylserine (PS) is required for the correct localization of Cdc42 [41] and Arl1 regulates the activity of the PS flippase Drs2 [42]. As we show that plasma membrane PS distribution is similar in WT and *arl1/arl1* cells, it is unlikely that *arl1/arl1* filamentous growth defect results from an alteration of PS distribution. Instead, we speculate that the inability of *arl1/arl1* to restrict hyphal growth to a single site is due to altered membrane traffic, as we observed an increase in protein secretion in this strain. Given that in *S*. *cerevisiae* membrane traffic is critical for Cdc42 function, *via* the delivery of this GTPase to sites of polarized growth [56–59], the increased membrane traffic in *C*. *albicans arl1/arl1* may promote competing sites of growth, which ultimately result in an inability to maintain growth to a single site. This effect may be more pronounced during hyphal growth due to increased membrane traffic. This work addresses for the first time the role of the Arf class of proteins in dimorphic fungi and identifies Arl1 and Arf2 as key regulators of *C*. *albicans* hyphal growth and virulence. Strikingly, we identify a unique role for Arl1 in the regulation of *C*. *albicans* secretion during filamentous growth. Investigating the role of Arl1 in filamentous fungi would determine if this role in secretion is unique to *C*. *albicans* or to the function in filamentous growth.

How are Arf2 and Arl1 regulated? Arf proteins do not have detectable intrinsic GTPase activity, hence the conversion of GTP to the GDP bound form requires a GAP (reviewed in [60]). In *S*. *cerevisiae*, Arf1 and Arl1 activities are regulated, in part, by the GAP Gcs1 [34]. In *C*. *albicans*, whether Arf2 and/or Arl1 are targets of the Gcs1 homolog, Age3 [27, 28] remains to be determined. In *S*. *cerevisiae*, as well as in mammals, Arf1 is activated by GEFs of the GBF/Gea1/2 and BIG/Sec7 families, at the early and late Golgi, respectively [6]. In *A*. *nidulans*, the Sec7 homolog HypB is critical for hyphal growth [61] and genetic analyses indicate that the HypB requirement can be bypassed by a single mutation in GeaA, which also results in the redistribution of GeaA towards the apical plasma membrane [62]. *S*. *cerevisiae* Sec7 itself is regulated by Arf1, *via* a positive feedback loop, as well as by Arl1 [63] similar to the situation in mammals, in which BIG1/2 is also regulated by Arf and Arl1 GTPases [64, 65]. Arl1 binds BIG1 *via* the N-terminal dimerization and cyclophilin binding (DCB) domain of this GEF in mammals [66], yet this is not the case for *S*. *cerevisiae* Sec7 [67]. In addition to Arf1 and Arl1, two Rab proteins, Ypt1 and Ypt31/32, the Rab1 and Rab11 homologs, respectively, play a role in recruiting Sec7 to the Golgi and, at least for Ypt31/32, further stimulating its activity [68]. Furthermore, Arl1 was shown to genetically interact with the Rab GTPase Ypt6 in *S*. *cerevisiae* [69, 70]. These examples highlight the complex crosstalk between Arf and Rab proteins and their regulators. In *C*. *albicans*, 3 out of the 5 *S*. *cerevisiae* GEF homologs are present, namely Sec7, Gea2 and Yel1. Intriguingly Syt1, which promotes Arl1 activation in *S*. *cerevisiae* [71, 72], has no homolog in *C*. *albicans*. Non-biaised approaches to identify Arl1 specific interactors during filamentous growth could lead to the identification of critical factors for *C*. *albicans* niche specific invasion.

## Materials and methods

### Growth conditions

Yeast extract-peptone dextrose (YEPD) or synthetic complete (SC) medium was used and strains were grown at 30°C, unless indicated otherwise. Filamentous growth induction was carried out as described previously either with 50% serum [73] or Spider medium [74]. Growth on YEPD plates containing Congo red, calcofluor white, caspofungin or fluconazole was examined as described [75]. Congo red, calcofluor white and Doxycycline were from Fluka, Sigma-Aldrich, Saint Quentin Fallavier, France. Caspofungin was from *Merck* Sharp & Dohme Corp.

### Strains and plasmids

Strains and oligonucleotides used are listed in S1 Table and S2 Table, respectively. All strains were derived from BWP17 [76]. The *arf1∆/arf1∆*, *arf3∆/arf3∆*, *arl1∆/arl1∆*, *arl3∆/arl3∆*, *imh1∆/imh1∆* and *drs2∆/drs2∆* strains were generated by homologous recombination. Each copy was replaced by either *HIS1* or *URA3*, using knockout cassettes generated by amplification of pGemHIS1 and pGemURA3 [76] with primer pairs ARF1.P1/ARF1.P2, ARF3.P1/ARF3.P2, ARL1.P1/ARL1.P2, ARL3.P1/ARL3.P2, IMH1.P1/IMH1.P2 and DRS2.P1/DRS2.P2. The *arl1∆/arl1∆/arl3∆/arl3∆* strain was generated from the *arl1∆/arl1∆*, in which each copy of *ARL3* was replaced by either *ARG4* or *SAT*, using knockout cassettes generated by amplification of pFAARG4 and pFASAT [77], respectively, with primer pairs ARL3.P5/ARL3.P6. The Dox repressible *arf1Δ/pTetARF1*, *arf2Δ/pTetARF2* and *arf3Δ/pTetARF3* strains were constructed from PY173, a derivative of BWP17 containing the tetracycline-regulatable transactivator TetR-ScHAP4AD, as described previously [43]. Specifically, the Tet_off_ promoter was inserted 5’ of one copy of *ARF1*, *ARF2* or *ARF3* ORF by homologous recombination using pCAU98 plasmid [78] as a template and either ARF1.P3/ARF1.P4, ARF2.P3/ARF2.P4 or ARF3.P3/ARF3.P4 primer pairs. The second copy of the respective ORF was then replaced with *HIS1* using a knockout cassette generated by amplification of pGemHIS1 with ARF1.P1/ARF1.P2, ARF2.P1/ARF2.P2 or ARF3.P1/ARF3.P2 primer pairs. *ARF2* and *ARL1* plasmids were constructed by amplification from gDNA. Primers with a unique XhoI (either ARF2.P5, ARL1.P3 or ARL3.P3) or SalI (DRS2.P3) at the 5’ end and a unique NotI at the 3’ end (either ARF2.P6, ARL1.P4, ARL3.P4 or DRS2.P4) were used to amplify the *ARF2*, *ARL1*, *ARL3* or *DRS2* ORF, together with 1 kb upstream and downstream, and the fragment subsequently cloned into pExpArg-pDCK1DCK1 [79], yielding to pExpArg-pARF2ARF2, pExpArg-pARL1ARL1 or pExpArg-pARL3ARL3 or pExpArg-pDRS2DRS2, respectively. Primers with a unique RsrII at the 5’ end (DRS2.P5) and a unique MluI at the 3’ end (DRS2.P6) were used to amplify the *DRS2* ORF, and the fragment subsequently cloned into pExpArgpADH-RAC1 [79], yielding to pExpArg-pADHDRS2.

pExpArg-pACT1GFPRID and pExpArg-pACT1CRIBGFP [40], as well as pExpArgpADHDRS2, were used to transform the *arl1/arl1* strain. pExpArgpARL1ARL1yemCherry was constructed by amplification of pARL1ARL1 from gDNA using ARL1.P5 and ARL1.P6, followed by integration of this fragment into the unique SacI and NotI sites in pExpArg-NotI-pADH1DCK1-SacI-yemCherry [80]. pExpArgpARL1arl1[G2A]yemCherry, pExpArgpARL1arl1[Q74L]yemCherry and pExpArgpARL1arl1[T34L]yemCherry were generated by site-directed mutagenesis of pExpArgpARL1ARL1yemCherry, using ARL1.P7/ARL1.P8, ARL1.P9/ARL1.P10 and ARL1.P11/ARL1.P12 primer pairs, respectively. pExpArgpACT1GFPIMH1_GRIP_ was constructed by amplification of the 513 bp encoding IMH1_GRIP_ fragment from gDNA using IMH1_GRIP_.P1 and IMH1_GRIP_.P2, followed by integration of this fragment into the unique RsrII and MluI sites in pExpArg-pACT1GFP-RsrII-RID-MluI [40]. Cdr1-GFP and FAPP1^[E50A,H54A]-^GFP expressing strains were generated as described [20] and the GFP-Sec4 and Sec7-GFP expressing strains were generated as described [44]. To visualize the distribution of phosphatidylserine, a fusion of GFP with the discoidin-like C2 domain of lactadherin was generated, similar that previously used [41]. Briefly, pExpArgpACT1GFPyeLactC2 was constructed by subcloning a synthesized 498 bp yeLactC2 DNA fragment, codon optimized for *C*. *albicans*, (Genescript, S2 Table), into the unique RsrII/MluI sites of pExpArgpACT1GFP-RsrIIRAC1MluI [79]. pDUP3pTEF1Phr2_SS_yemCherry*-PHR2* was constructed similar to that of *S*. *cerevisiae* [81] and will be described elsewhere (P. de Oliveira e Silva, M. Bassilana and R. A. Arkowitz, in preparation).

All pExpArg plasmids were linearized with StuI and integrated into the RP10 locus. Two independent clones of each strain were generated, confirmed by PCR as well as immunobloting, where relevant. All PCR amplified products and site-directed mutagenesis products were confirmed by sequencing (Eurofins MWG Operon, Ebersberg, Germany).

### Microscopy analyses

For colony morphology analyses, plates were incubated for 3–6 days prior to imaging. For cell morphology studies, cells were imaged by differential interference contrast. Cell viability was determined using propidium iodide staining [82].

For Arl1-yemCherry, GFP-Sec4 and Sec7-GFP imaging, z-stacks were acquired approximately every min, as described [20]. For Arl1 and Sec7 co-localization experiments, GFP and yemCherry signals were acquired simultaneously. The number of Golgi cisternae that had both GFP and yemCherry signals was quantitated using Volocity. Multiple z-sections were acquired over 5 times and averages were from the intersection of GFP and yemCherry signals from 10 fields of view (*n* ~ 5 cells per field of view; ~500 punctae in total). Maximum intensity projections of 21 z-sections were generated with ImageJ software. GFPRID and CRIBGFP distribution experiments were carried out as described [40]. Unless indicated otherwise, error bars represent the standard deviation. Golgi and colocalization analyses were carried out as described [20].

### Virulence assays

To induce haematogenously disseminated candidiasis (HDC), 8 Balb/C mice per strain were injected *via* the tail vein with an inoculum of 5x10^5^ cells [83]. Oropharyngeal candidiasis (OPC) was induced according to established procedure [84]. Results were analyzed using the Wilcoxon rank sum test.

### Ethics statement

All mouse experiments were approved by the Animal Care and Use Committee at the Los Angeles Biomedical Research Institute and carried out according to the National Institutes of Health (NIH) guidelines for the ethical treatment of animals (protocols 011000 and 012059; IACUC # A3330-01).

### General techniques

Western blot analyses were carried out as described previously [73]. For visualization, membranes were probed with an Alexa Fluor 800 anti-rabbit IgG (H+L) conjugate (1:10,000; Molecular Probes, Invitrogen) and visualized using an Odyssey IR imaging system (LI-COR Biosciences). The actin cytoskeleton was visualized and imaged as described previously [43]. FM4-64 labeling was carried out as in [28]. RT-PCR and qRT-PCR analyses were carried out as described [80, 85], and the primers used (GENE.pTm and GENE.mTm) are listed in S2 Table or previously described [85]. For transcriptome profiles, RNA extraction was carried out using a Master Pure Yeast RNA purification kit (Epicentre) and the samples analyzed at the Genecore facility (EMBL, Heidelberg). Analyses of secreted Hwp1_ss_-GFP were carried out as described [20]. Genomic DNA from *C*. *albicans* strains was isolated as described in [86]. For Southern analyses, EcoRI digested gDNA was separated on a 1% agarose gel, transferred onto a nylon membrane, and fixed by UV crosslinking. Southern hybridization was carried out with ECL labeled probes (generated by PCR with ARF2.P10 and ARF2.P11 primers), as described in the Amersham ECL Direct Nucleic Acid Labelling and Detection System kit (GE Healthcare UK Limited, Little Chalfont Buckinghamshire, UK).

## Supporting information

S1 Table Strains used in the study. (PDF)

S2 Table Primer sequences. (PDF)

S1 Fig Sequence alignments of *C*. *albicans* Arf/Arl proteins. A) Sequence alignments of *C*. *albicans* Arf1-3 and Arl1. Identities in 2 or more sequences are shown in black and similarities in grey. Consensus indicates identities in all 4 sequences. B) Sequence alignment of *C*. *albicans* Arl3 with its *S*. *cerevisiae* and Human (ARFRP1) counterparts. Identities in 2 or more sequences are shown in black and similarities in grey.
(PDF)

S2 Fig Arf/Arl mutant strains verification. A) Diagram of *Δ/pTetARF2* strain construction. Primers used to verify the *Δ/pTetARF2* strain by PCR are indicated, as well as the EcoRI sites used for the Southern analyses. B) PCR analyses of *Δ/pTetARF2* mutant. The absence of *pARF2ARF2*, in the *Δ/pTetARF2* strain, was verified using primer 2 in the ORF (ARF2.P7) and primer 1 in pARF2 (ARF2.P9), which amplifies a 670 bp fragment from the endogenous copy in WT and complemented strains. The knock-in of the *URA3*-pTet cassette was verified using two primer pairs: primers 1 (ARF2.P9) and 6 in *URA3* (URA3.P1) and primers 2 (ARF2.P7) and 7 in pTet (TET.P1), to generate DNA fragments each of 450 bp. The replacement of one copy of *ARF2* by *HIS1* was verified using two primer pairs: primers 1 (ARF2.P9) and 3 in *HIS1* (HIS1.P1) and primers 4 in *ARF2* terminator region (ARF2.P8) and 5 in *HIS1* (HIS1.P2), to generate DNA fragments of 650 bp and 1060 bp, respectively. C) Southern blot. The EcoRI digested gDNA from the indicated strains was separated on a 1% agarose gel. Southern hybridization, visualized with a ECL labeled probe (generated by PCR with ARF2.P10 and ARF2.P11), revealed in the *Δ/pTetARF2* strain the presence of the expected 2.9 kb and 5.9 kb fragments and the absence of the endogenous 3.7 kb fragment (present in the WT strain); an additional fragment corresponding to the integrated copy of *ARF2* at the RP10 locus is observed in the *Δ/pTetARF2* complemented strain. D) *ARF2* transcript levels. Transcript levels of *ARF2* were determined by qRT-PCR in the indicated strains, using ARF2.pTm/ARF2.mTm primer pair and normalized to the *ACT1* transcript level. The mean values of two independent experiments are shown with bars indicating values of each experiment. E) *ARF1*, *ARF3* and *ARL1* transcript levels. mRNA and cDNA were prepared from the indicated strains. *ARF1*, *ARF3* and *ARL1* transcripts were determined by RT-PCR, using ARF1.pTm/ARF1.mTm (82 bp), ARF3.pTm/ARF3.mTm (99 bp) and ARL1.pTm/ARL1.mTm (99 bp) primer pairs, respectively. Actin (*ACT1*) transcript levels (ACT1.pTm/ACT1.mTm primer pair) were used for normalization. F) *ARL3* transcript levels. *ARL3* expression was assessed in the indicated strains, as described in E, using ARL3.pTm/ARL3.mTm primer pair to generate a 116 bp fragment. G) *ARF2* expression is higher than that of the other *ARF/ARL* genes. The relative gene expression of two biological samples of the WT strain, grown in the absence of FCS induction, is the read counts per gene normalized by the ORF size, determined by RNAseq.
(PDF)

S3 Fig Arf2 is critical for antifungal sensitivity. A) Repressible expression of *ARF2* confers thermosensitivity. Serial dilutions of cells from the indicated strains were spotted on YEPD media. Images were taken after 2 days growth at the indicated temperature. B) The *Δ/pTetARF2* mutant has increased susceptibility to a variety of stresses, including antifungal drugs. Serial dilutions of indicated strains were spotted on YEPD media containing 400 μg/ml Congo red (CR), 25 μg/ml calcofluor white (CFW), 5 μg/ml fluconazole (Fluco) or 125 ng/ml caspofungin (Caspo). Images were taken after 2 days. Similar results were observed in 2 experiments. C) Distribution of the multidrug ABC transporter Cdr1 is not altered in *Δ/pTetARF2* and *arl1/arl1* cells. DIC and deconvolved central z-section of representative WT, *Δ/pTetARF2* and *arl1/arl1* cells expressing Cdr1-GFP grown in the absence of Dox.
(PDF)

S4 Fig Reintroduction of *ARF2* and *ARL1* complements *Δ/pTetARF2* and *arl1/arl1* mutants, respectively. A) Reintroduction of *ARF2* restores *Δ/pTetARF2* viability. Serial dilutions of indicated strains were spotted on YEPD media with or without 20 μg/ml Dox. B) Reintroduction of *ARF2* complements for *Δ/pTetARF2* invasive growth defect. Cells from the indicated strains were incubated, in the absence of Dox, on agar-containing Spider media (top) or YEPD media containing FCS (bottom) and images were taken after 5 days. C) Reintroduction of *ARF2* complements for *Δ/pTetARF2* cell wall integrity defect. Serial dilutions of the indicated strains were spotted on YEPD media containing 400 μg/ml Congo red (CR) or 25 μg/ml calcofluor white (CFW). Images were taken after 2 days. D) Reintroduction of *ARF2* complements for *Δ/pTetARF2* antifungal hypersensitivity. Serial dilutions of the indicated strains were spotted on YEPD media containing the indicated concentrations of caspofungin or fluconazole and images were taken after 2 days; *Δ/pTetARF2* has a MIC of 30 ng/ml and 600 ng/ml for caspofungin and fluconazole, respectively. E) Reintroduction of *ARL1* complements *arl1/arl1* cell wall integrity defect. Serial dilutions of the indicated strains were spotted on YEPD media containing CR or CFW, as in S4C Fig. F) Reintroduction of *ARL1* complements the *arl1/arl1* invasive growth defect. Cells from the indicated strains were incubated as in S4B Fig and images were taken after 5 days. G) The Arl1-yemCherry fusion is functional. Cells from the indicated strains were incubated on Spider media and images were taken after 5 days. Similar results were observed in 2 independent experiments.
(PDF)

S5 Fig Arl1 is required for site-specific infection. A) The *arl1/arl1* mutant has a decreased virulence in OPC. Histopathology images of the tongues, stained with periodic acid-Schiff (PAS), of the mice infected with the indicated strains as in 2B. B) The *arl1/arl1* mutant has an increased tropism for the brain. In the HDC assay, the fungal burden of lateral tail vein infected Balb/C mice, with the WT and *arl1/arl1* strains, was measured in the indicated organs after 2 days of infection. Results are expressed as in Fig 2B.
(PDF)

S6 Fig Drs2, but not Imh1 is required for cell wall integrity. A) *IMH1* transcript levels. *IMH1* expression was assessed in the indicated strains, as described in S2E Fig, using IMH1.pTm/IMH1.mTm primer pair to generate a 144 bp fragment. B) Imh1 is not required for cell wall integrity. Serial dilutions of the indicated strains were spotted on YEPD media containing CR or CFW, as in S4C Fig. C) *DRS2* transcript levels. *DRS2* expression was assessed in the indicated strains, as in S2E Fig, using DRS2.pTm/DRS2.mTm primer pair to generate a 65 bp fragment. D). The *drs2/drs2* mutant is hypersensitive to fluconazole. Serial dilutions of indicated strains were spotted on YEPD media containing or lacking CR, CFW or Fluco, as in S3B Fig. *The drs2/drs2* mutant has a MIC of 300 ng/ml for fluconazole, as estimated using conditions in S4D Fig.
(PDF)
