# Supplementary material for: Role of Arf GTPases in fungal morphogenesis and virulence
Source: PLoS Pathog. 2017 Feb 13;13(2):e1006205. doi: 10.1371/journal.ppat.1006205 (PMC5325608; doi:10.1371/journal.ppat.1006205)
Supplement: S1 Table — (PDF) [file ppat.1006205.s001.pdf]

S1 Table: Strains used in the study.

STRAIN	RELEVANT GENEOTYPE	REFERENCE
BWP17	*ura3Δ::λimm434/ura3Δ::λimm434 his1Δ::hisG/his1Δ::his arg4::hisG/arg4Δ::hisG*	[76]
PY82	*ura3Δ::λ imm434/ura3Δ::λ imm434 his1::hisG/HIS1::his1::hisG arg4::hisG/URA3::ARG4::arg4::hisG*	[73]
PY173	*ade2Δ::hisG/ade2Δ::hisG ura3Δ::λimm434/ura3Δ::λimm434 his1Δ::hisG/his1Δ::hisG arg4Δ::hisG/arg4Δ::hisG ENO1/eno1::ENO1-tetR ScHAP4AD-3×HA-ADE2*	[43]
PY977	Same as PY173 with *HIS1 URA3::ARG4::arg4::hisG*	[43]
PY1927	Same as PY173 with *ARF1/ arf1::URA3pTetoffARF1*	This study
PY1930	Same as PY173 with *ARF2/ arf2::URA3pTetoffARF2*	This study
PY1980	Same as PY173 with *arf1Δ::HIS1/ arf1::URA3pTetoffARF1*	This study
PY1984	Same as PY173 with *arf2Δ::HIS1/ arf2::URA3pTetoffARF2*	This study
PY2014	Same as PY1980 with *RP10::ARG4*	This study
PY2015	Same as PY1984 with *RP10::ARG4*	This study
PY2200	Same as BWP17 with *CDR1/CDR1::CDR1-GFP-SAT1*	[20]
PY2330	Same as BWP17 with *arf3Δ::HIS1/ ARF3*	This study
PY2347	Same as BWP17 with *arf3Δ::HIS1/ arf3Δ::URA3*	This study
PY2355	Same as PY173 with *arf3Δ::HIS1/ ARF3*	This study
PY2360	Same as PY1984 with *RP10::ARG4 pADHFAPP1$^{[E50A,H54A]}$GFP*	This study
PY2363	Same as PY173 with *arf3Δ::HIS1/ arf3::URA3pTetoffARF3*	This study
PY2381	Same as PY2347 with *RP10:: ARG4 pARF3ARF3*	This study
PY2383	Same as PY2347 with *RP10::ARG4*	This study
PY2384	Same as PY2363 with *RP10::ARG4*	This study
PY2436	Same as PY173 with *arf2Δ::HIS1/ ARF2* and *RP10::URA3-ARG4*	This study
PY2496	Same as PY2015 with *CDR1/CDR1::CDR1-GFP-SAT1*	This study

PY2504	Same as BWP17 with *arl1Δ::HIS1/ ARL1*	This study
PY2533	Same as BWP17 with *arl1Δ::HIS1/ arl1Δ::URA3*	This study
PY2538	Same as PY2533 with *RP10::ARG4*	This study
PY2552	Same as PY2533 with *RP10:: ARG4 pARL1ARL1*	This study
PY2578	Same as BWP17 with *RP10::ARG4 pADHFAPP1$^{[E50A,H54A]}$GFP*	[20]
PY2606	Same as PY2533 with *RP10:: ARG4 pACT1CRIBGFP*	This study
PY2607	Same as PY2533 with *RP10:: ARG4 pACT1GFPRID*	This study
PY2613	Same as PY2533 with *RP10::ARG4 pADHFAPP1$^{[E50A,H54A]}$ GFP*	This study
PY2643	Same as BWP17 with *RP10::ARG4- pADH1Hwp1$_{ss}$GFP*	[20]
PY2723	Same as PY1984 with *RP10::ARG4 pARF2ARF2*	This study
PY2739	Same as BWP17 with *RP10::ARG4- pACT1GFP-Imh1$_{GRIP}$*	This study
PY2745	Same as PY2533 with *RP10::ARG4 pACT1GFP-Imh1$_{GRIP}$*	This study
PY2760	Same as BWP17 with *arf1Δ::HIS1/ ARF1*	This study
PY2765	Same as PY2533 with *CDR1/CDR1::CDR1-GFP-SAT1*	This study
PY2781	Same as BWP17 with *arf1Δ::HIS1/ arf1Δ::URA3*	This study
PY2818	Same as PY2781 with *RP10::ARG4*	This study
PY2866	Same as BWP17 *with arl3Δ::HIS1/ ARL3*	This study
PY2875	Same as BWP17 with *arl3Δ::HIS1/ arl3Δ::URA3*	This study
PY2878	Same as PY2875 with *RP10::ARG4*	This study
PY3029	Same as 2533 with *RP10::ARG4- pADH1Hwp1$_{ss}$GFP*	This study
PY3073	Same as PY2875 with *RP10::ARG4 pARL3ARL3*	This study
PY3091	Same as PY2875 with *RP10::ARG4 pACT1GFP-Imh1$_{GRIP}$*	This study
PY3175	Same as BWP17 with *RP10::ARG4 pARL1ARL1-yemCherry*	This study
PY3177	Same as PY2533 with *RP10::ARG4 pARL1ARL1-yemCherry*	This study
PY3179	Same as PY3175 with *SEC4/SEC4::GFP-*	This study

	SEC4-URA3	
PY3226	Same as PY2875 with *RP10::ARG4 pARL1ARL1-yemCherry*	This study
PY3231	Same as PY3175 with *SEC7/SEC7::SEC7-GFPɤ-URA3*	This study
PY3239	Same as BWP17 with *RP10::ARG4 pACT1GFP-yeLactC2*	This study
PY3242	Same as PY2533 with *RP10::ARG4 pACT1GFP-yeLactC2*	This study
PY3303	Same as BWP17 with *drs2Δ::HIS1/ DRS2*	This study
PY3310	Same as BWP17 with *drs2Δ::HIS1/ drs2Δ::URA3*	This study
PY3362	Same as PY3310 with *RP10::ARG4 pACT1GFP-yeLactC2*	This study
PY3374	Same as PY3310 with *RP10::ARG4 pDRS2DRS2*	This study
PY3375	Same as PY3310 with *RP10::ARG4*	This study
PY3439	Same as BWP17 *with imh1Δ::HIS1/ IMH1*	This study
PY3441	Same as BWP17 with *imh1Δ::HIS1/ imh1Δ::URA3*	This study
PY3445	Same as PY3441 with *RP10::ARG4*	This study
PY3497	Same as PY2533 with *RP10::ARG4 pADH1DRS2*	This study
PY3583	Same as BWP17 with *NEU5L::SAT-pTEF1- Phr2$_{SS}$yemCherry-PHR2*	De Oliveira e Silva, Bassilana & Arkowitz, in prep.
PY3586	Same as PY2533 with *NEU5L::SAT-pTEF1- Phr2$_{SS}$yemCherry-PHR2*	This study
PY3649	Same as PY2533 with *RP10::ARG4 pARL1arl1[Q74L]-yemCherry*	This study
PY3651	Same as PY2533 with *RP10::ARG4 pARL1arl1[G2A]-yemCherry*	This study
PY3666	Same as PY2533 with *RP10::ARG4-pARL1arl1[T34L]-yemCherry*	This study
PY3708	Same as PY2533 with *arl3Δ::ARG4/ ARL3*	This study
PY3783	Same as PY3708 with *arl3Δ::ARG4/ arl3Δ::SAT*	This study
