# Supplementary material for: Role of Arf GTPases in fungal morphogenesis and virulence
Source: PLoS Pathog. 2017 Feb 13;13(2):e1006205. doi: 10.1371/journal.ppat.1006205 (PMC5325608; doi:10.1371/journal.ppat.1006205)
Supplement: S2 Table — (PDF) [file ppat.1006205.s002.pdf]

S2 Table: Primer sequences

PRIMER	SEQUENCE
ARF1.P1	GGCACATAATCTTTCGGTTCACACAAAATCAACTGATTTTTTGTCTAACAGCTCTAATCAAGTATAGTTATCTACTGTGGAATTGTGAGCGGATA
ARF1.P2	CAACCACATTTTATATTATACTTACTTTACATAACCCAAAATACCGGAGATAATACTCGAGGGGGCACGGAGATTTCTTTCCCAGTCACGACGTT
ARF1.P3	GTTGTCGTACTTCTGGTCGTGTACCATTCCAAACTGTTCACACTTTTTTTAGGTCATGGCACATAATCTTTCGGTTCACACAGGAATTGATTTGGATGG
ARF1.P4	GCATCCAAACCAACCATCAATATTCTCATTTCACGTCTCCCCAAGAGACTGGCAAATAATTTTGAAATTGTTAATCCCATCTAGTTTTCTGAGATAAAGCTG
ARF2.P1	CTGTATTTTATAACTGTTATCAGTTCCACTTGTTCATTTATCAAAAAAGAAAAAATATTGTTGTAATTAGTCATGGGTGTGGAATTGTGAGCGGATA
ARF2.P2	GAAAGACAACAAAAGAACAAAACTTAACTTAATTTAATTTGACTTAACTTAACTTAACTTAATCCATTTATGAAGAGTTTCCCAGTCACGACGTT
ARF2.P3	CAATCATAAAAGAATCATCTATCTTTAATTTTTTTTAGCATTACTAGATTTTCAGATAAAAACTGTATTTTATAACTGTTATCAGAGGAATTGATTTGGATGG
ARF2.P4	GCATCTAAACCAACCATCAAGATTCTCATTTCTTTGTTACCAAAGAGATTAGCAAATAATTTGGAAAATGATAAACCCATCTAGTTTTCTGAGATAAAGCTG
ARF2.P5	ATCTCGAGTATGCAGTATTTCTTCGGGG
ARF2.P6	TATAGCGGCCGCAATTGATACTACCAGAAAAA
ARF2.P7	CTCTAGCTTCAGCAATACG
ARF2.P8	GGCTAGAGTTAATGTTTTCTG
ARF2.P9	CAACGAAAGCAACCCAACTT
ARF2.P10	GTTCCCACTTCACCACTTC
ARF2.P11	CACAACAATAGGCAATTGTCC
ARF3.P1	CCTTCCTGTCCTTCATTCGTTCAATATAGATAAACACATCAACTGTCGTTCGAAATGGGTGGTTTAGTGGTATGTGTGGAATTGTGAGCGGATA
ARF3.P2	CGTGTGTATCTCTTTCCCTCGCCCTCTCTCTTAGTTTAGATTATTTATATGTATGTTTAAGGTAAGATTTACTTGGTTTCCCAGTCACGACGTT
ARF3.P3	CCTCAATTTTTAGACGAGAATTTAGACAACTCATTCAACTTTTCCATTCATTTATTTGTGAATTTCCTTCCTGTCCAGGAATTGATTTGGATGG
ARF3.P4	AAAATATAGTTTGTTAAAGAAAAAACAATCATAATGAATATGAATGACATACCACTAAACCACCCATCTAGTTTTCTGAGATAAAGCTG
ARF3.P5	ATCTCGAGTTTTCAGTGTCGACTTCTAC
ARF3.P6	TATAGCGGCCGCGAATTAGCAACACTAAAAC
ARL1.P1	TCACTAACAGGAACAACGACATCCATAAACAGTATTATATAAACAACATTTTATTCAGCAAATAATTACATGTGGAATTGTGAGCGGATA
ARL1.P2	CTAACTTCTGATGACATTTTTTTATTTTTATTACATTTCGATTCTTATTCTATACAGTTACGAAAATACTATCCGCTATCACTTTCCCAGTCACGACGTT
ARL1.P3	ATCTCGAGATTTGTTTCTGAAGTTGCCGG
ARL1.P4	TATAGCGGCCGCGCCACTGACAAATCGTTTG
ARL1.P5	TCGAGCTCAAACCAGCACCGTCACCCAACTGCTCATCCTTAATTACATCC
ARL1.P6	TATAGCGGCCGCATTTGTTTCTGAAGTTGCCGG
ARL1.P7	CAGCAAATAATTACCATGGCTCAAGCTTTTAGTTTTGG
ARL1.P8	CCAAAACTAAAAGCTTGAGCCATGGTAATTATTTGCTG

ARL1.P9	GGGATTTGGGTGGACTGACGTCCATTAGACCTTAC
ARL1.P10	GTAAGGTCTAATGGACGTCAGTCCACCCAAATCCC
ARL1.P11	CTAGATGGTGCTGGTAAGCTTACAATATTGTATCGTTTG
ARL1.P12	CAAACGATACAATATTGTAAGCTTACCAGCACCATCTAG
ARL3.P1	CAGATACTGTGTTCCATTCCATTCAATTTGCTCCTAACCGATATTCGTACCTT ATAATTTGCATTTTAAATGTTTCTGTGGAATTGTGAGCGGATA
ARL3.P2	CTATTTTCGAAAGTATCGTAAATGTGTTAATTGCGATTCTATTGTATTTAATC TCTATTTTCACTTATACTCAGGCTTTCCCAGTCACGACGTT
ARL3.P3	ATCTCGAGCAATTGTCTTTCAAGATGTTGG
ARL3.P4	TATAGCGGCCGCCTATGACGGTGCCAAATTAATAC
ARL3.P5	GATACTGTGTTCCATTCCATTCAATTTGCTGCTAACCGATATTCGTACCTTAT AATTTGCATTTTAAATGTTTCATCGAAGCTTCGTACGCTGCAGGTC
ARL3.P6	AAAAACTATTTTCGAAAGTATCGTAAATGTGTTAATTGCGATTCTATTGTAT TTAATCTCTATTTTCACTTATACTCTGATATCATCGATGAATTCGAG
DRS2.P1	CATAATTTTTTGTTGTTGTTTTTTTATTAAAGGCTGTGTTGTCTAATAAAACA TTGACAATGTCCAATTATAATCGGTGTGGAATTGTGAGCGGATA
DRS2.P2	CATTCATTGATTGCATTAATATACTACTCATACTCTATTTACTTACTTTCTGAT AATTCCCCAAAAACTCCTCTTTCCCAGTCACGACGTT
DRS2.P3	ATGTCGACCTTTTAATATAGCCGAATTCCCCG
DRS2.P4	TATAGCGGCCGCGAGTTGGTTAATAACAAAATATCAG
DRS2.P5	TACGGACCGATGTCCAATTATAATCGGACAGATAA
DRS2.P6	ATACGCGTTTACTTACTTTCTGATAATTCCCCAAAAAC
IMH1.P1	ATTGAAAAAATAAAGAACTACCTTGAAGCACAATTTCAACTATCAAGAGAA ATCAAATGTTTTCGAAACTTAAGAATGTGGAATTGTGAGCGGATA
IMH1.P2	CTTTGCTTGGGTGATAACTCGCACTACCAAGTGGATAATAATACATTAGTTA CTACTCATTTCAATGCCATTAAGAACTTTCCCAGTCACGACGTT
TET.P1	CAACAGAGAAAGGGCAGG
URA3.P1	GGCACTACAGCAACTTTC
HIS1.P1	CTTTAGATGGATCGACATTGG
HIS1.P2	AGTCCTCGATGTTGTCG
$IMH1_{GRIP}$.P1	GCACGGACCGTGTCAGTTCAAGAGTACAAATTGAAAGTGG
$IMH1_{GRIP}$.P2	GATACGCGTCATTTCAATGCCATTAAGAACTTTTCTTCATC
yeLactC2	GCACGGACCGTGTGTACTGAACCATTAGGTTTAAAAGATAATACTATTCCA AATAAACAAATTACTGCTTCATCATATTATAAAACTTGGGGTTTGTCAGCTT TTTCATGGTTTCCATATTATGCTAGATTAGATAATCAAGGTAAATTTAATGCT TGGACTGCTCAAACTAATTCAGCTTCAGAATGGTTGCAAATTGATTTAGGTT CACAAAAAAGAGTTACTGGTATTATTACTCAAGGTGCTAGAGATTTTGGTC ATATTCAATATGTTGCTGCTTATAGAGTTGCTTATGGTGATGATGGTGTTA CTTGGACTGAATATAAAGATCCAGGTGCTTCAGAATCAAAAATTTTTCCAGG TAATATGGATAATAATTCACATAAAAAAAATATTTTTGAAACTCCATTTCAA GCTAGATTTGTTAGAATTCAACCAGTTGCTTGGCATAATAGAATTACTTTGA GAGTTGAATTATTGGGTTGTTAAACGCGTACG
RT.PCR primers	
ACT1.pTm	ATGTTCCCAGGTATTGCTGA
ACT1.mTm	ACATTTGTGGTGAACAATGG

ARF1.pTm	CCGACCACTATGGCGCTAC
ARF1.mTm	CGTTGATTCTGTCCCGGTC
ARF2.pTm	CCGCTGAAATAACTGAAAAATTAGG
ARF2.mTm	AGTAGATAACCATTCCAAACCTTCGT
ARF3.pTm	GGGATTGTGGTGGTCAAGAAA
ARF3.mTm	CTGTCAACATCCAGGGAATCAA
ARL1.pTm	CTGGAGATGTTACTACAGC
ARL1.mTm	CATTTGATGTAATTCCTTG
ARL3.pTm	ATCCTCCACGGAATCAGCAA
ARL3.mTm	CCAACTGTGGGTAAAATCCGTTTA
IMH1.pTm	GGAGGAGGGCTCGGAAA
IMH1.mTm	GATAGGTGCCGGAAGTTGAT
DRS2.pTm	GCGGCATTAGTGGTTACCAT
DRS2.mTm	AGCAGGATACCAACCTAACCAT
