# Supplementary material for: Role of Arf GTPases in fungal morphogenesis and virulence
Source: PLoS Pathog. 2017 Feb 13;13(2):e1006205. doi: 10.1371/journal.ppat.1006205 (PMC5325608; doi:10.1371/journal.ppat.1006205)
Supplement: S1 Fig — A) Sequence alignments of C. albicans Arf1-3 and Arl1. Identities in 2 or more sequences are shown in black and similarities in grey. Consensus indicates identities in all 4 sequences. B) Sequence alignment of C. albicans Arl3 with its S. cerevisiae and Human (ARFRP1) counterparts. Identities in 2 or more sequences are shown in black and similarities in grey. (PDF) [file ppat.1006205.s003.pdf]

A

```
CaArf1       1  MG--LTISKLFASLLG-RREMRILMVGLDAAGKTTILYKLKLGEIVTTIPTIGFNVETVE
CaArf2       1  MG--LSFSKLFANLFG-NKEMRILMVGLDAAGKTTILYKLKLGEIVTTIPTIGFNVETVE
CaArl1       1  MGQAFSFGNIFSKLWGTNKEIRILILGLDGAGKTTILYRLQMGEVVTTKPTIGFNVETLK
CaArf3       1  MG------GLVSKLFK-NREMRILMLGLDNAGKTTILYKLKLGKTSKTVPTVGFNVETVK
consensus    1  **  ....... .*.. ..*.***..***.*******.*..*....*.**.******.

CaArf1      58  YKNISFTVWDVGGQDKIRPLWRYYFQNTQGIIFVVDSNDRDRINEAREELQSMLNEDELK
CaArf2      58  YKNISFTVWDVGGQDKIRPLWRYYFQNTQGIIFVVDSNDRDRIAEAREELQQMLNEDELR
CaArl1      61  YKNITLNIWDLGGQTSIRPYWRCYYSNTSAVIFVVDSTDKDRIDTACKELHQMLKEEELQ
CaArf3      54  HKNVSFAVWDCGGQERIRPLWRHYFTGTNALIYVVDSSDVDRLEESKQELFRIVTDKELT
consensus   61  .**.....**.***..***.**.*...*...*.****.*.**......**.......**.

CaArf1     118  DAVLLVLANKQDLPNAMNAAEITEKMGLHSIR-NRPWFIQATCATTGDGLYEGLEWLSNQ
CaArf2     118  DALLLVFANKQDLPNAMNAAEITEKLGLHSIR-QRPWYIQATCATTGDGLYEGLEWLSTN
CaArl1     121  DSALLVFANKQDQPGAMTAAEVSQALSLTDLK-DRSWSIVASSAIKGEGLTEGLDWLMDV
CaArf3     114  NCLLVVLANKQDVDGAVKPKDLIERFQLNKLTGEHTWSVIPTIAIDGTGLVETLNWISSH
consensus  121  ...*.* *****.. *...........*....  ..*......* .*.**.*.*.*....

CaArf1     177  VGK---  179
CaArf2     177  LKNSS-  181
CaArl1     180  IKDEQL  185
CaArf3     174   SK---- 175
consensus  181  ...   .*
```

B

```
CaArl3     1  MFHLASSLYTQYTKREQYNILILGLDNAGKTTFLEHLKLLYSSSTESANKNQLSKNSTTS
ScArl3     1  MFHLVKGLYNNWNKKEQYSILILGLDNAGKTTFLETLKKEYSLAFKALEK----------
HsARFP1    1  MYTLLSGLYKYMFQKDEYCILILGLDNAGKTTFLEQSKTRFNKNYKGMSLS---------

CaArl3    61  DDQPRSTSDVIKSKRILPTVGQNTTTIKFESKDSDSPLASQFKNINLKFWDLGGQKSLRN
ScArl3    51  ---------------IQPTVGQNVATIPVDSKQ------------ILKFWDVGGQESLRS
HsARFP1   52  --------------KITTTVGLNIGTVDVGKAR------------LMFWDLGGQEELQS

CaArl3   121  MWSRYFKQCHGIIFIIDSTDTERFQECYETLIDIAHDDIWMQIDDDDNDNDNSNVDGTV
ScArl3    84  MWSEYYSLCHGIIFIVDSSDRERLDECSTTLQSVVMD----------------EEIEG--
HsARFP1   85  LWDKYYAECHGVIYVIDSTDEERLAESKQAFEKVVTS----------------EALCG--

CaArl3   181  NVPILMMANKQDLPQAVDLVSLKTGVFIKLVSELEATDSKLLPVSVLENQGLQESLEWLV
ScArl3   126  -VPILMLANKQDRQDRMEVQDIKE-VFNKIAEHISARDSRVLPISALTGEGVKDAIEWMI
HsARFP1  127  -VPVLVLANKQDVETCLSIPDIKT-AFSDCTSKIGRRDCLTQACSALTGKGVREGIEWMV

CaArl3   241  TRLIYNKRNKKPEYK---
ScArl3   184  VRLERNKKSRPPIYK---
HsARFP1  185  KCVVRN-VHRPPRQRDIT
```
