# Supplementary figures and images for: Role of Arf GTPases in fungal morphogenesis and virulence
Source: PLoS Pathog. 2017 Feb 13;13(2):e1006205. doi: 10.1371/journal.ppat.1006205 (PMC5325608; doi:10.1371/journal.ppat.1006205)

**A**

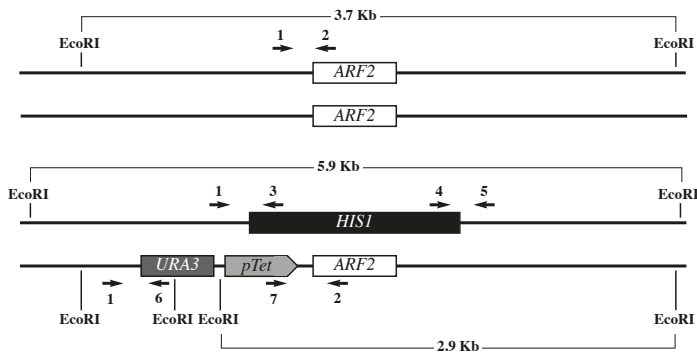

**B**

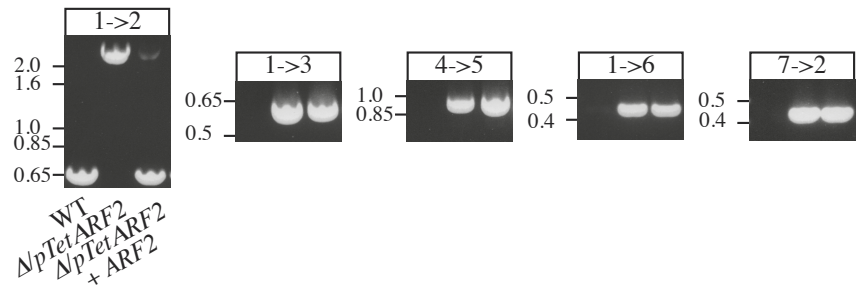

**C**

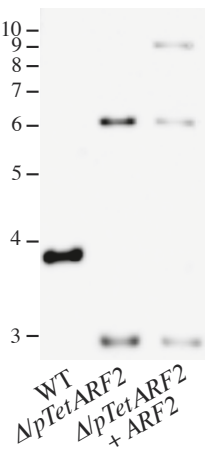

**D**

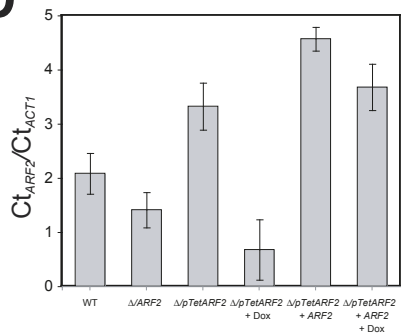

**E**

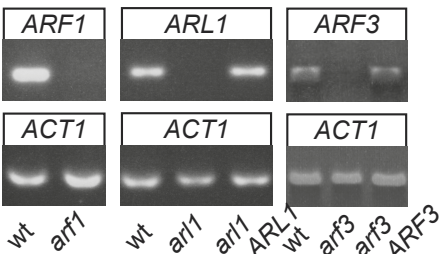

**F**

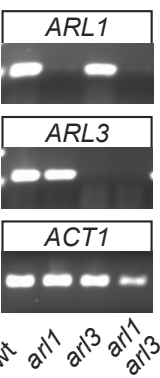

**G**

| Gene | Rel. Exp. 1 | Rel. Exp. 2 | Avg. Rel. Exp. |
|------|-------------|-------------|----------------|
| ARF1 | 0.77        | 0.55        | 0.66           |
| ARF2 | 9.33        | 11.76       | 10.54          |
| ARF3 | 1.06        | 0.81        | 0.93           |
| ARL1 | 1.11        | 1.28        | 1.20           |
| ARL3 | 0.86        | 0.78        | 0.82           |
| ACT1 | 19.63       | 21.52       | 20.58          |

Supplement: S2 Fig — A) Diagram of Δ/pTetARF2 strain construction. Primers used to verify the Δ/pTetARF2 strain by PCR are indicated, as well as the EcoRI sites used for the Southern analyses. B) PCR analyses of Δ/pTetARF2 mutant. The absence of pARF2ARF2, in the Δ/pTetARF2 strain, was verified using primer 2 in the ORF (ARF2.P7) and primer 1 in pARF2 (ARF2.P9), which amplifies a 670 bp fragment from the endogenous copy in WT and complemented strains. The knock-in of the URA3-pTet cassette was verified using two primer pairs: primers 1 (ARF2.P9) and 6 in URA3 (URA3.P1) and primers 2 (ARF2.P7) and 7 in pTet (TET.P1), to generate DNA fragments each of 450 bp. The replacement of one copy of ARF2 by HIS1 was verified using two primer pairs: primers 1 (ARF2.P9) and 3 in HIS1 (HIS1.P1) and primers 4 in ARF2 terminator region (ARF2.P8) and 5 in HIS1 (HIS1.P2), to generate DNA fragments of 650 bp and 1060 bp, respectively. C) Southern blot. The EcoRI digested gDNA from the indicated strains was separated on a 1% agarose gel. Southern hybridization, visualized with a ECL labeled probe (generated by PCR with ARF2.P10 and ARF2.P11), revealed in the Δ/pTetARF2 strain the presence of the expected 2.9 kb and 5.9 kb fragments and the absence of the endogenous 3.7 kb fragment (present in the WT strain); an additional fragment corresponding to the integrated copy of ARF2 at the RP10 locus is observed in the Δ/pTetARF2 complemented strain. D) ARF2 transcript levels. Transcript levels of ARF2 were determined by qRT-PCR in the indicated strains, using ARF2.pTm/ARF2.mTm primer pair and normalized to the ACT1 transcript level. The mean values of two independent experiments are shown with bars indicating values of each experiment. E) ARF1, ARF3 and ARL1 transcript levels. mRNA and cDNA were prepared from the indicated strains. ARF1, ARF3 and ARL1 transcripts were determined by RT-PCR, using ARF1.pTm/ARF1.mTm (82 bp), ARF3.pTm/ARF3.mTm (99 bp) and ARL1.pTm/ARL1.mTm (99 bp) primer pairs, respectively. Actin (AC [file ppat.1006205.s004.pdf]

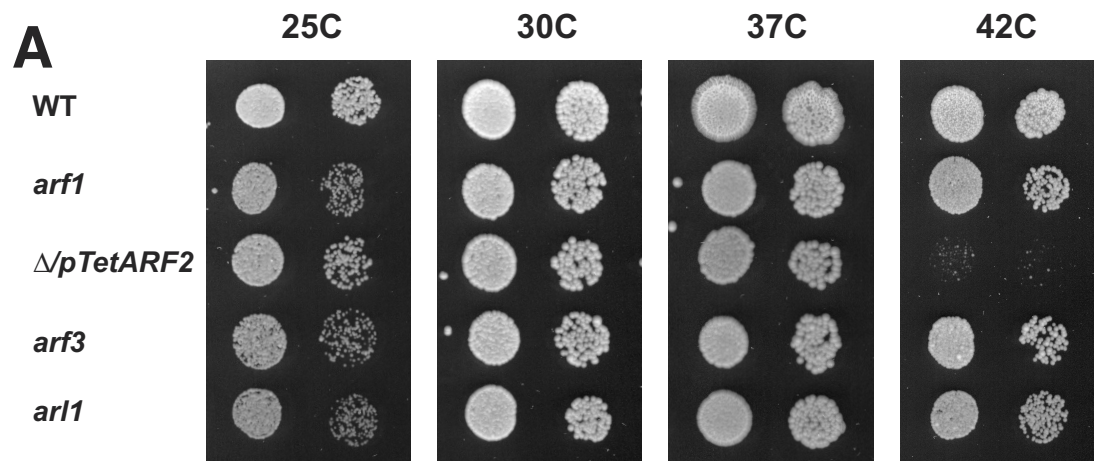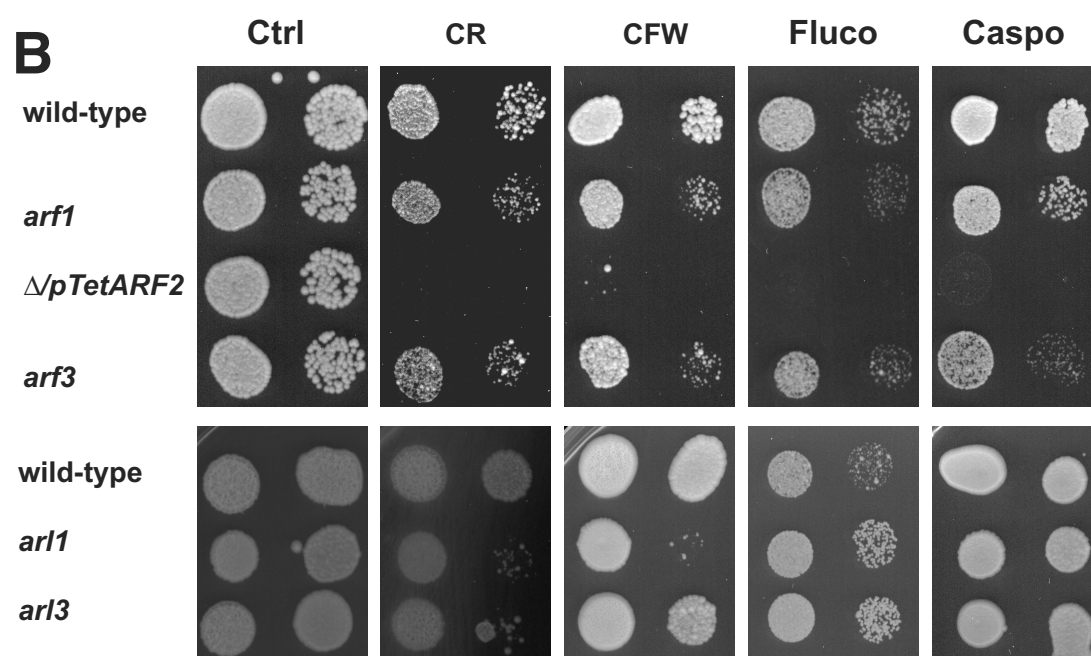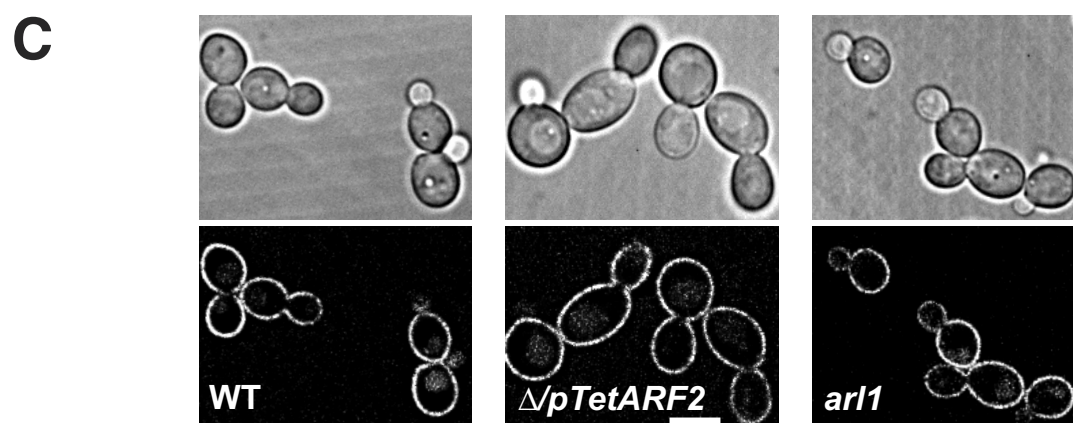

Supplement: S3 Fig — A) Repressible expression of ARF2 confers thermosensitivity. Serial dilutions of cells from the indicated strains were spotted on YEPD media. Images were taken after 2 days growth at the indicated temperature. B) The Δ/pTetARF2 mutant has increased susceptibility to a variety of stresses, including antifungal drugs. Serial dilutions of indicated strains were spotted on YEPD media containing 400 μg/ml Congo red (CR), 25 μg/ml calcofluor white (CFW), 5 μg/ml fluconazole (Fluco) or 125 ng/ml caspofungin (Caspo). Images were taken after 2 days. Similar results were observed in 2 experiments. C) Distribution of the multidrug ABC transporter Cdr1 is not altered in Δ/pTetARF2 and arl1/arl1 cells. DIC and deconvolved central z-section of representative WT, Δ/pTetARF2 and arl1/arl1 cells expressing Cdr1-GFP grown in the absence of Dox. (PDF) [file ppat.1006205.s005.pdf]

**A**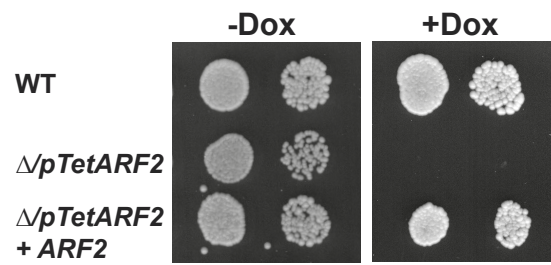**B**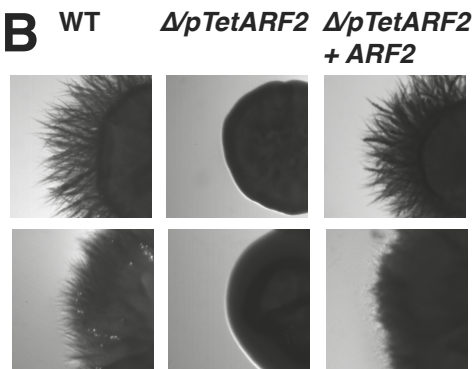**C**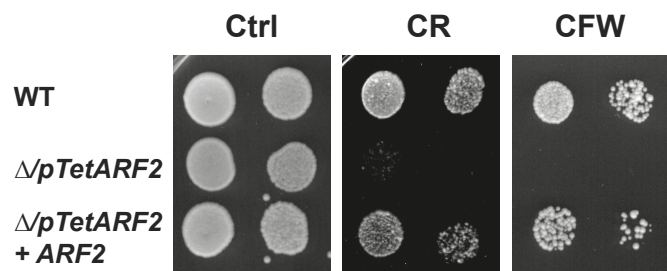**D**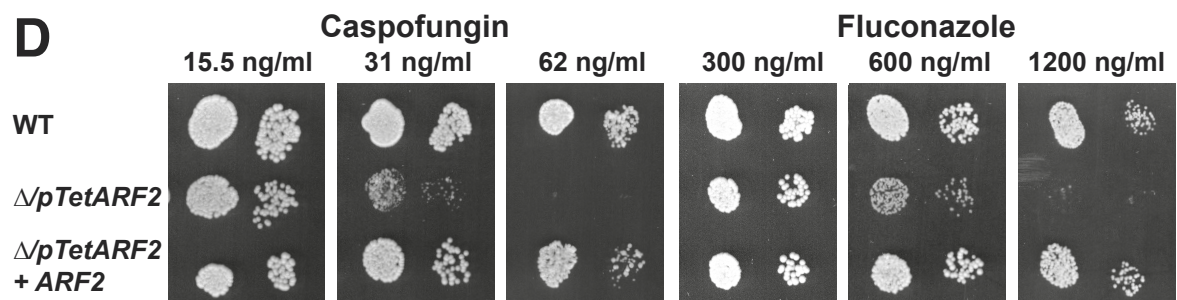**E**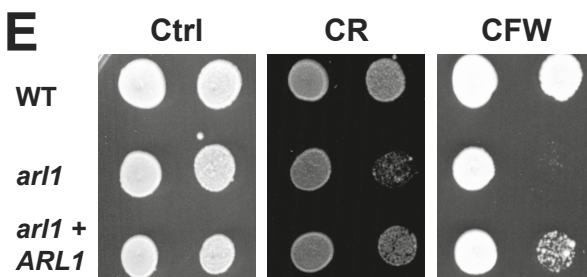**F**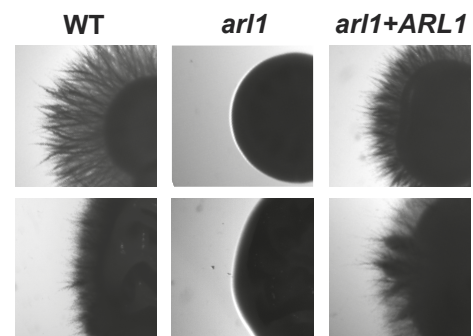**G**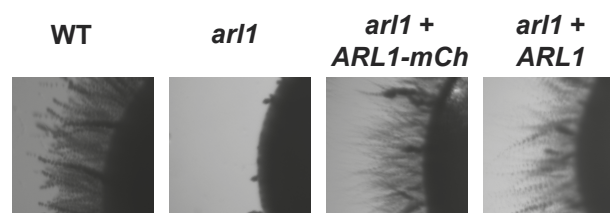

Supplement: S4 Fig — A) Reintroduction of ARF2 restores Δ/pTetARF2 viability. Serial dilutions of indicated strains were spotted on YEPD media with or without 20 μg/ml Dox. B) Reintroduction of ARF2 complements for Δ/pTetARF2 invasive growth defect. Cells from the indicated strains were incubated, in the absence of Dox, on agar-containing Spider media (top) or YEPD media containing FCS (bottom) and images were taken after 5 days. C) Reintroduction of ARF2 complements for Δ/pTetARF2 cell wall integrity defect. Serial dilutions of the indicated strains were spotted on YEPD media containing 400 μg/ml Congo red (CR) or 25 μg/ml calcofluor white (CFW). Images were taken after 2 days. D) Reintroduction of ARF2 complements for Δ/pTetARF2 antifungal hypersensitivity. Serial dilutions of the indicated strains were spotted on YEPD media containing the indicated concentrations of caspofungin or fluconazole and images were taken after 2 days; Δ/pTetARF2 has a MIC of 30 ng/ml and 600 ng/ml for caspofungin and fluconazole, respectively. E) Reintroduction of ARL1 complements arl1/arl1 cell wall integrity defect. Serial dilutions of the indicated strains were spotted on YEPD media containing CR or CFW, as in S4C Fig. F) Reintroduction of ARL1 complements the arl1/arl1 invasive growth defect. Cells from the indicated strains were incubated as in S4B Fig and images were taken after 5 days. G) The Arl1-yemCherry fusion is functional. Cells from the indicated strains were incubated on Spider media and images were taken after 5 days. Similar results were observed in 2 independent experiments. (PDF) [file ppat.1006205.s006.pdf]

**A**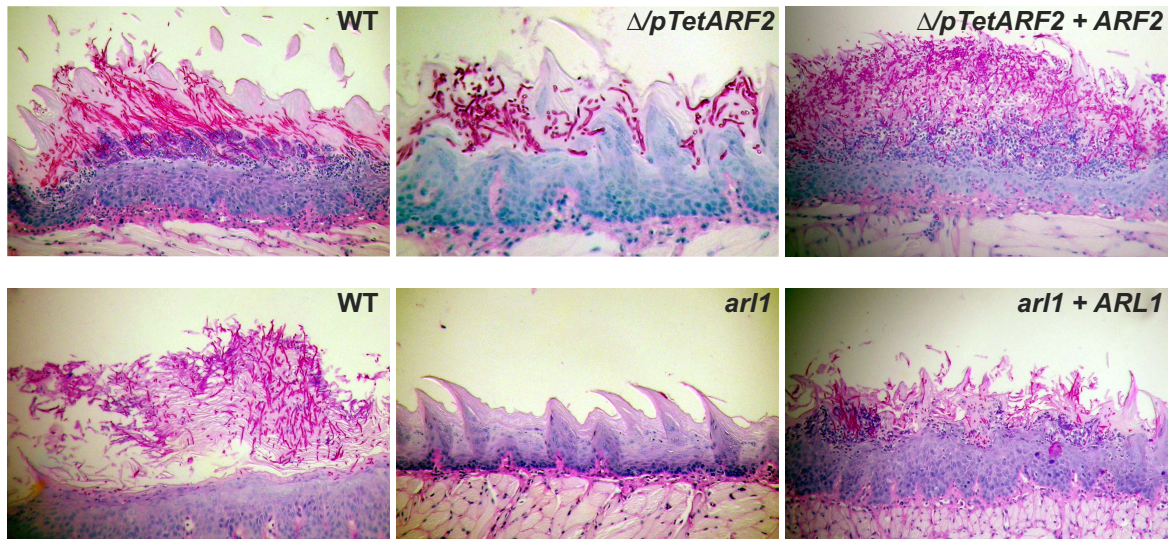**B**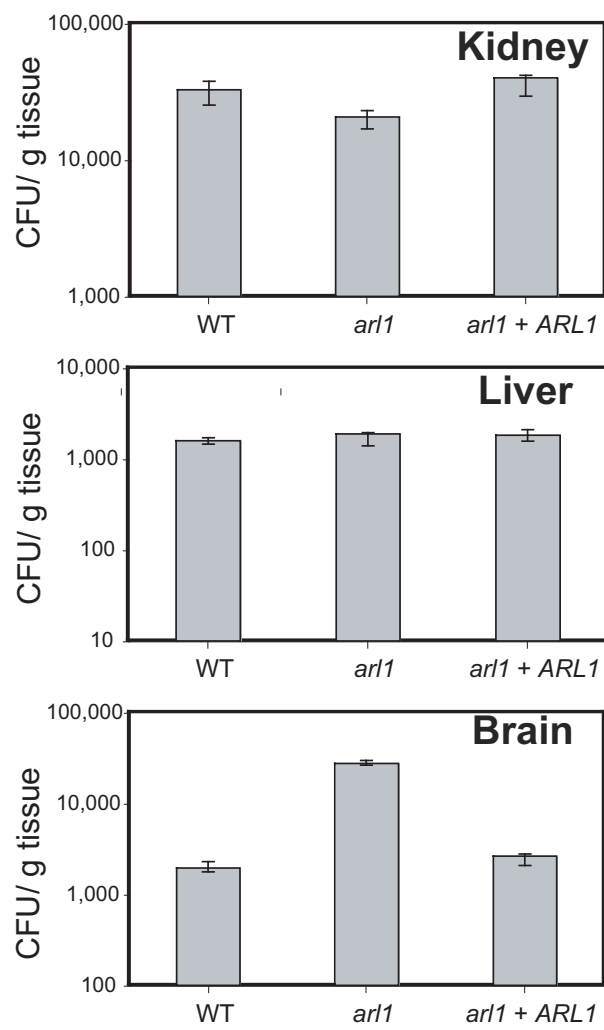

Supplement: S5 Fig — A) The arl1/arl1 mutant has a decreased virulence in OPC. Histopathology images of the tongues, stained with periodic acid-Schiff (PAS), of the mice infected with the indicated strains as in 2B. B) The arl1/arl1 mutant has an increased tropism for the brain. In the HDC assay, the fungal burden of lateral tail vein infected Balb/C mice, with the WT and arl1/arl1 strains, was measured in the indicated organs after 2 days of infection. Results are expressed as in Fig 2B. (PDF) [file ppat.1006205.s007.pdf]

**A**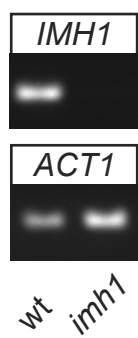**B**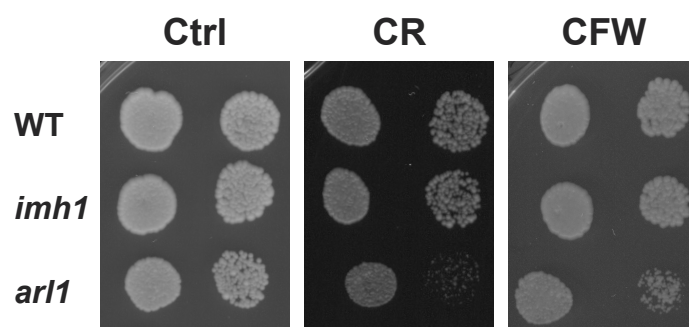**C**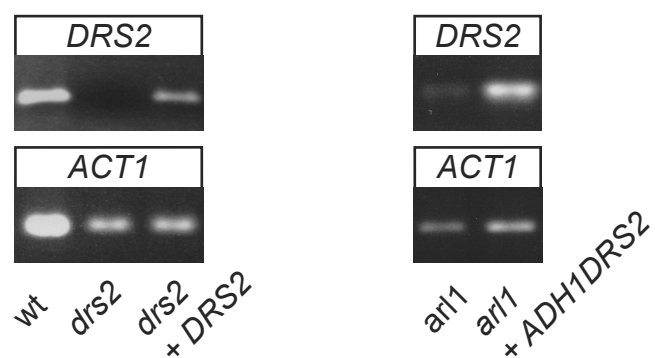**D**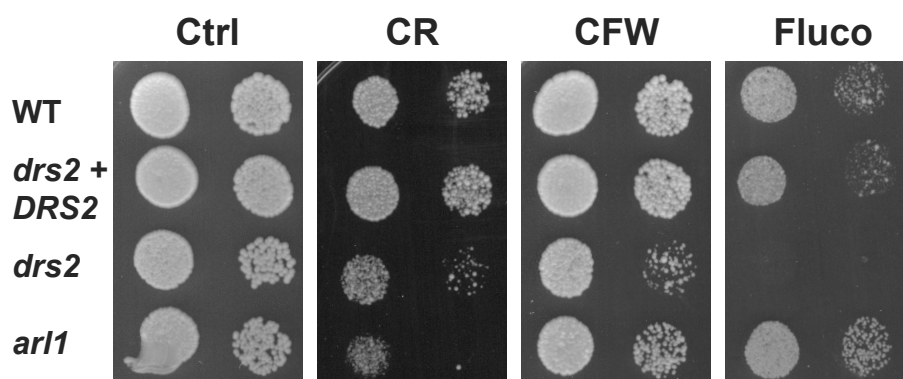

Supplement: S6 Fig — A) IMH1 transcript levels. IMH1 expression was assessed in the indicated strains, as described in S2E Fig, using IMH1.pTm/IMH1.mTm primer pair to generate a 144 bp fragment. B) Imh1 is not required for cell wall integrity. Serial dilutions of the indicated strains were spotted on YEPD media containing CR or CFW, as in S4C Fig. C) DRS2 transcript levels. DRS2 expression was assessed in the indicated strains, as in S2E Fig, using DRS2.pTm/DRS2.mTm primer pair to generate a 65 bp fragment. D). The drs2/drs2 mutant is hypersensitive to fluconazole. Serial dilutions of indicated strains were spotted on YEPD media containing or lacking CR, CFW or Fluco, as in S3B Fig. The drs2/drs2 mutant has a MIC of 300 ng/ml for fluconazole, as estimated using conditions in S4D Fig. (PDF) [file ppat.1006205.s008.pdf]
